# Random Transitions of a Binary Star in the Canonical Ensemble

**DOI:** 10.3390/e26090757

**Published:** 2024-09-04

**Authors:** Pierre-Henri Chavanis

**Affiliations:** Laboratoire de Physique Théorique, Université de Toulouse, CNRS, UPS, 31062 Toulouse, France; chavanis@irsamc.ups-tlse.fr

**Keywords:** statistical mechanics, self-gravitating systems, ensemble inequivalence, metastable states, random transitions, Fokker–Planck equation, Kramers formula

## Abstract

After reviewing the peculiar thermodynamics and statistical mechanics of self-gravitating systems, we consider the case of a “binary star” consisting of two particles of size *a* in gravitational interaction in a box of radius *R*. The caloric curve of this system displays a region of negative specific heat in the microcanonical ensemble, which is replaced by a first-order phase transition in the canonical ensemble. The free energy viewed as a thermodynamic potential exhibits two local minima that correspond to two metastable states separated by an unstable maximum forming a barrier of potential. By introducing a Langevin equation to model the interaction of the particles with the thermal bath, we study the random transitions of the system between a “dilute” state, where the particles are well separated, and a “condensed” state, where the particles are bound together. We show that the evolution of the system is given by a Fokker–Planck equation in energy space and that the lifetime of a metastable state is given by the Kramers formula involving the barrier of free energy. This is a particular case of the theory developed in a previous paper (Chavanis, 2005) for *N* Brownian particles in gravitational interaction associated with the canonical ensemble. In the case of a binary star (N=2), all the quantities can be calculated exactly analytically. We compare these results with those obtained in the mean field limit N→+∞.

## 1. Introduction

Self-gravitating systems have very peculiar thermodynamics and statistical mechanics because of the unshielded long-range attractive nature of the gravitational force.^1^ As a consequence, self-gravitating systems exhibit peculiar features:

(i) A violent collisionless relaxation leading to the formation of a quasistationary (or metaequilibrium) state whose lifetime diverges when the number of particles N→+∞;
(ii) A slow collisional relaxation leading to a long-lived metastable state (in idealized situations) whose lifetime increases exponentially with *N*;
(iii) The absence of a statistical equilibrium state in a strict sense due to the evaporation of high-energy stars and the phenomenon of core collapse (gravothermal catastrophe or isothermal collapse);
(iv) The inequivalence of statistical ensembles due to the nonadditivity of energy and entropy implying, for example, the existence of negative specific heats;^2^
(v) The occurrence of zeroth- and first-order phase transitions between a dilute phase and a condensed phase when a small-scale regularization has been introduced in the system. The existence of long-lived metastable states can lead to a hysteresis behavior associated with cycles of gravitational “collapse” and “explosion” at the spinodal points.

These curious behaviors (or some of them) also occur in other systems interacting via long-range forces such as two-dimensional point vortices in hydrodynamics, the Hamiltonian mean-field (HMF) model, and non-neutral plasmas. It is therefore important to develop a general framework to treat systems with long-range interactions and describe the numerous analogies (and sometimes the differences) between them. In that respect, self-gravitating systems provide a model of fundamental interest for which ideas of statistical mechanics and thermodynamics can be tested and developed. The main domain of application is astrophysics, but general methods can be transposed to other fields of physics.

The microcanonical ensemble is the correct description of Hamiltonian stellar systems such as globular clusters [10,15,17]. Apart from slow evaporation, these systems can be assumed isolated in a first approximation, so they conserve mass and energy. Since energy is non-additive, the canonical ensemble is not justified to describe the statistics of a sub-part of these systems. However, the canonical ensemble may be relevant for astrophysical systems in contact with a thermostat of non-gravitational origin.^3^ In particular, this is the correct description of a gas consisting of self-gravitating Brownian particles [22]. This system is dissipative, so it evolves at fixed mass and temperature, instead of fixed mass and energy. Brownian particles interact via self-gravity, but they also experience a friction force and a stochastic force (noise), which mimic the interaction of the system with an external medium to which the particles are coupled through short-range interactions.^4^ In this model, the temperature measures the coupling with the bath. It is thus of conceptual interest to describe the two systems (Hamiltonian and Brownian) in parallel in order to show the analogies and the differences between a microcanonical (fixed *E*) and a canonical (fixed *T*) evolution.

In principle, to determine the statistical equilibrium state of a physical system, we have to compute the density of states g(E) in the microcanonical ensemble and the partition function Z(β) in the canonical ensemble. For self-gravitating systems, these two quantities diverge. A first reason is due to the long-distance behavior of the potential of interaction and the fact that the system has the tendency to evaporate. This is already the case for an ordinary gas if it is not confined in a container. To simplify the discussion, we shall consider the case of a self-gravitating system enclosed within a spherical box of radius *R*. The case of an unbounded domain requires nonequilibrium statistical mechanics and kinetic theory and will be briefly discussed in the conclusion of this paper. Even when confined in a box, the density of states and the partition function diverge because of the short-distance behavior of the potential of interaction and the fact that the system has the tendency to collapse. We can make the density of states and the partition function finite by introducing a small-scale regularization. For example, a small-scale cut-off may correspond to the size *a* of the particles.^5^ With these cut-offs (a,R) dealing with UV and IR divergences, the density of state and the partition function are finite. However, they cannot be evaluated analytically in general. We thus have to find particular situations where the calculation can be simplified.

A first possibility is to consider a proper thermodynamic limit N→+∞ with $ER/GM^{2}∼1$, βGMm/R∼1 and a/R∼1 (see, e.g., Appendix A of [1] for details). In this limit, the mean field approximation is exact except close to a critical point. Furthermore, we can make a connection between statistical mechanics and thermodynamics. Indeed, one can show that for N→+∞, the density of states g(E) in the microcanonical ensemble is dominated by the macrostate that maximizes the Boltzmann entropy S[f] at fixed mass *M* and energy *E*. Similarly, in the canonical ensemble, the partition function Z(β) is dominated by the macrostate that minimizes the Helmholtz free energy F[f]=E[f]−TS[f] at fixed mass *M*. We can then determine the series of equilibria that contains all the extrema of entropy at fixed mass and energy or, equivalently, all the extrema of free energy at fixed mass.^6^ This includes fully stable, metastable, and unstable states. The strict caloric curve is made of fully stable states. However, for systems with long-range interactions, metastable states are very long-lived because their lifetime scales as $e^{N}$ [29]. Therefore, it is important to take them into account. As a result, the physical caloric curve should contain stable and metastable states. We can then describe phase transitions between dilute and condensed states and study the regions where the statistical ensembles are equivalent or inequivalent. In particular, the region of negative specific heat in the microcanonical ensemble is replaced by a phase transition in the canonical ensemble. The series of equilibria of the self-gravitating gas and the nature of phase transitions in microcanonical and canonical ensembles depend on the small-scale regularization *a* or, equivalently, on the size *R* of the system. This problem has been studied in detail in [15] in the case of self-gravitating fermions.^7^ The system displays canonical and microcanonical phase transitions and exhibits two critical points, one in each ensemble, at which the phase transition disappears. This is similar to the critical point in the classical van der Waals gas. For large systems, there exist microcanonical and canonical phase transitions. For intermediate systems, there exist only canonical phase transitions. For small systems, there is no phase transition at all. If we first take the limit N→+∞, then the limit a→0, the physical caloric curve contains only long-lived metastable states and displays situations of gravitational collapse in the microcanonical and canonical ensembles, which are called gravothermal catastrophe [17] and isothermal collapse [40], respectively.

Another tractable example is the case of N=2 particles of size *a* in gravitational interaction in a spherical box of radius *R* [10,15]. This provides a toy model of self-gravitating systems. For this “binary star” model, it is possible to compute the density of states and the partition function exactly analytically and show explicitly the difference between a microcanonical and a canonical description. Interestingly, this simple model already displays many features of the true *N*-body problem with a large number of particles. It illustrates the notion of ensemble inequivalence and the fact that the region of negative specific heat in the microcanonical ensemble is replaced by a first-order phase transition in the canonical ensemble. It can also be used to describe random transitions of the system between a “dilute” state where the particles are well separated and a “condensed” state where the particles are bound together.

This paper is organized as follows. In Section 2, we discuss the statistical mechanics of isolated self-gravitating systems in the microcanonical ensemble. In Section 3, we discuss the statistical mechanics of dissipative self-gravitating Brownian particles in the canonical ensemble. In each case, we give special attention to the mean field limit N→+∞ and point out the importance of metastable states. In Section 4, we study the fluctuations of energy in the canonical ensemble. In Section 5, we study the lifetime of metastable states in the canonical ensemble by using a Fokker–Planck approach and making connections with the Kramers formula. In Section 6, we determine the normal form of the thermodynamical potential close to the critical temperature and simplify the expression of the metastable state lifetime. In Section 7, we study the dynamical evolution of the energy in the canonical ensemble. We determine the relaxation time above the critical temperature and the collapse time below the critical temperature. We consider finite-size scalings at the critical point. In Section 8, we study the statistical mechanics of a binary star in the microcanonical ensemble. In Section 9, we study the statistical mechanics of a binary star in the canonical ensemble. In Section 10, we study the fluctuations of the energy of a binary star in the canonical ensemble. In Section 11, we study the random transitions of a binary star between a “dilute” state and a “condensed” state in the canonical ensemble. In Section 12, we determine the lifetime of a binary star with a=0 in the canonical ensemble.

**Remark** **1.** *Preliminary results of the binary star (N=2) model have been published in Ref. [15]. The present paper provides a much deeper and more complete analysis of this model and details the calculations of the metastable state lifetime close to the critical point.*

## 2. Isolated Self-Gravitating Systems in the Microcanonical Ensemble

### 2.1. Hamilton Equations

We consider a system of *N* particles with mass *m* in Newtonian gravitational interaction. The particles can be stars in a stellar system (like a galaxy or a globular cluster) or atoms in a self-gravitating gas. Their dynamics are described by the Newton equations of motion, which can be cast in a Hamiltonian form

(1) $$\begin{array}{c} m\frac{dr_{i}}{dt}=\frac{\partial H}{\partial v_{i}},\;m\frac{dv_{i}}{dt}=-\frac{\partial H}{\partial r_{i}},\\ H=\frac{1}{2}\sum_{i=1}^{N}mv_{i}^{2}+U\left(r_{1},\ldots ,r_{N}\right). \end{array}$$

In the following, we shall consider the gravitational potential of interaction $U=-\sum_{i<j}Gm^{2}/\left|r_{i}-r_{j}\right|$ in d=3 dimensions. However, the general formalism of statistical mechanics remains valid for an arbitrary potential of interaction *U*.

### 2.2. Microcanonical Distribution

We assume that the system is isolated so that there is conservation of mass and energy. Therefore, the correct statistical ensemble is the microcanonical ensemble and the control parameter is the energy *E*. The evolution of the *N*-body distribution function $P_{N}\left(r_{1},v_{1},\ldots ,r_{N},v_{N},t\right)$ is governed by the Liouville equation [20,21]

(2) $$\frac{\partial P_{N}}{\partial t}+\sum_{i=1}^{N}\left(v_{i}\cdot \frac{\partial P_{N}}{\partial r_{i}}+F_{i}\cdot \frac{\partial P_{N}}{\partial v_{i}}\right)=0,$$

where $F_{i}=-\frac{1}{m}\partial U/\partial r_{i}$ is the total force (by unit of mass) experienced by particle *i*. For t→+∞, the system is expected to achieve a statistical equilibrium state described by the microcanonical distribution

(3) $$\begin{array}{c} P_{N}\left(r_{1},v_{1},\ldots ,r_{N},v_{N}\right)=\frac{1}{g(E)}\delta \left(E-H\right). \end{array}$$

This distribution states that, for an isolated system, all accessible microstates (with energy *E*) are equiprobable. This is the fundamental postulate of equilibrium statistical mechanics. The normalization factor of the distribution function

(4) $$\begin{array}{c} g\left(E\right)=\int \delta \left(E-H\right)\prod_{i=1}^{N}dr_{i}dv_{i} \end{array}$$

gives the density of states with energy *E*. This is the quantity of fundamental interest in the microcanonical ensemble. The entropy is defined as follows:^8^

(5) S(E)=lng(E).

The temperature is then given by

(6) $$\frac{1}{T(E)}=S^{\prime }\left(E\right).$$

The temperature is a function of energy. The function T(E) defines the microcanonical caloric curve. By differentiating the foregoing relation we obtain

(7) $$\begin{array}{c} S^{\prime \prime }\left(E\right)=-\frac{T^{\prime }\left(E\right)}{T^{2}\left(E\right)}. \end{array}$$

This relation can be written as

(8) $$\begin{array}{c} S^{\prime \prime }\left(E\right)=-\frac{1}{T^{2}\left(E\right)C\left(E\right)}, \end{array}$$

where

(9) $$\begin{array}{c} C\left(E\right)=\frac{1}{T^{\prime }\left(E\right)}=\frac{dE}{dT} \end{array}$$

is the microcanonical specific heat. In the microcanonical ensemble, the specific heat can be positive or negative. When it is negative, we have $S^{\prime \prime }\left(E\right)>0$ so the entropy curve S(E) presents a convex intruder.

**Remark** **2.** *One of the most striking properties of self-gravitating systems is the existence of negative specific heats. Astronomers have used this concept for a long time [17,41]. It is indeed well known that if radiation energy is extracted from a star whose nuclear fuel is exhausted, the star will contract and heat up. The negative specific heat of self-gravitating systems is a consequence of the virial theorem. Consider a self-gravitating system in a steady state. According to the virial theorem, the kinetic energy K and the potential energy W are related to each other by the the following relation:^9^*

(10) 2K+W=0.

*For an isothermal system $K=\frac{3}{2}NT$. Therefore, the total energy reads*

(11) $$E=K+W=-K=-\frac{3}{2}NT$$

*and the specific heat is negative:*

(12) $$C_{V}=\frac{dE}{dT}=-\frac{3}{2}N<0.$$

*By losing heat, the system grows hotter and continues to radiate energy. This is the mechanism that prevails in the internal region of stars. For instance, at a stage where no more nuclear fuel is available, the core contracts and becomes hotter, giving its energy to the outer part which expands and becomes colder. Thus, the process continues until the star has reached very high densities.^10^ Beckenstein [42] and Hawking [43] have demonstrated that black holes also have negative specific heats and display the same phenomenon.*

### 2.3. Divergences at Short and Large Distances

For self-gravitating systems in d=3 dimensions, if the domain is unlimited, the density of states diverges when the distribution of particles is spread to infinity [10]. This is already the case for an ordinary gas if it is not confined in a container. Therefore, there is no equilibrium state in a strict sense. Stellar systems have the tendency to evaporate.^11^ In practice, a stellar system such as a globular cluster is subject to the tides of a nearby galaxy. Therefore, it only extends over a region of size *R*, say, where *R* is the tidal radius above which the stars escape. We can account heuristically for this confinement by considering the statistical mechanics of stellar systems confined within a spherical box of radius *R* against which the stars bounce elastically.

Even when the particles are confined within a box, the statistical mechanics of self-gravitating systems is rather subtle. Indeed, for N≥3, the density of states (4) diverges when two stars approach each other at infinitely close distances, while the released energy is redistributed among the other stars (halo) in the form of kinetic energy [10]. This shows the natural tendency of stellar systems to form binary stars [44,45,46]. A binary star surrounded by a hot halo can indeed be viewed as the strict equilibrium state of a stellar system in the microcanonical ensemble. However, there may exist other statistical equilibrium states in the form of gaseous configurations where the stars are at large distances from each other. These states correspond to robust metastable equilibrium states with very long lifetimes scaling as $e^{N}\;t_{D}$ [29]. They only exist at sufficiently high energies. Most globular clusters are described by metastable states that are not yet collapsed [47].

In order to regularize the divergence of the density of states at small separations and make the problem well defined mathematically, we can use a softening potential or consider the case of hard spheres with size *a*.^12^ Note that for N=2, the density of states (4) exists even without a small-scale cut-off (i.e., when a=0). Indeed, in the argument used to show the divergence of the density of states, we need at least a third particle (N≥3) to absorb the potential energy released by the binary.

### 2.4. Thermodynamic Limit N→+∞

For systems with long-range interactions, we can define a proper thermodynamic limit N→+∞ with $ER/GM^{2}∼1$, βGMm/R∼1 and a/R∼1 in which the system is extensive but remains fundamentally nonadditive. In this limit, we can make a mean field approximation and develop a thermodynamic approach. Indeed, in the microcanonical ensemble, we can write the density of states g(E) under the form

(13) $$\begin{array}{c} g\left(E\right)=\int e^{Ns[f]}\delta \left(E-E\left[f\right]\right)\delta \left(M-M\left[f\right]\right)Df, \end{array}$$

where S[f]=Ns[f] is the entropy and the integral in Equation (13) runs over all the possible distribution functions f(r,v) that yield the correct mass M=∫ρdr and energy $E=\frac{1}{2}\int fv^{2}\;drdv+\frac{1}{2}\int \rho \Phi \;dr$ (where ρ=∫fdv is the density and Φ the gravitational potential).^13^ It is important to note that for systems with long-range interactions, the entropy is proportional to *N*. As a result, in the thermodynamic limit N→+∞, the integral (13) is dominated by the macrostate that maximizes the entropy at fixed mass and energy. This leads to the thermodynamical principle

(14) $$\begin{array}{c} S\left(E\right)=\max_{f}\left\{S[f]\;|\;M,E\;\mathrm{fixed}\right\}, \end{array}$$

which determines the most probable equilibrium state in the microcanonical ensemble. This can be viewed as a result of large deviations.

In order for g(E) to be finite, we must introduce a small-scale regularization. We have two possibilities. (i) If we consider classical pointlike particles, the entropy is the Boltzmann entropy:

(15) $$\begin{array}{c} S\left[f\right]=Ns\left[f\right]=-\int \frac{f}{m}\ln\frac{f}{m}\;drdv. \end{array}$$

It can be obtained by a standard combinatorial analysis [51]. In order for g(E) to be finite, we must introduce a softening length *a* in the gravitational potential by using for example the Plummer potential $U=-\sum_{i<j}Gm^{2}/\sqrt{|r_{i}-r_{j}{|}^{2}+a^{2}}$. (ii) Alternatively, if we use the original gravitational potential $U=-\sum_{i<j}Gm^{2}/\left|r_{i}-r_{j}\right|$ (without softening), in order for g(E) to be finite, we must take into account the size *a* of the particles. In that case, we must change the form of the entropy. One can use, for example, the Ornstein [52] generalized entropy, which is associated with the van der Waals equation of state (see, e.g., [53]). We could also consider the case of fermions and use the Fermi–Dirac entropy in phase space [28] or configuration space [39]. The study of gravitational phase transitions in these different situations, depending on the value of *a*, is discussed in [15,28,39]. In the following, to simplify the discussion, we shall consider the limit a→0. Therefore, we use the Boltzmann entropy (15) and the original gravitational potential of interaction $U=-\sum_{i<j}Gm^{2}/\left|r_{i}-r_{j}\right|$. In that case, the density of state g(E) diverges but the maximization problem (14) may still have a solution corresponding to a long-lived metastable state.

### 2.5. Metastable States

Let us recall the well-known results concerning the maximization problem (14) for self-gravitating point masses (a=0).

**Figure 1:**
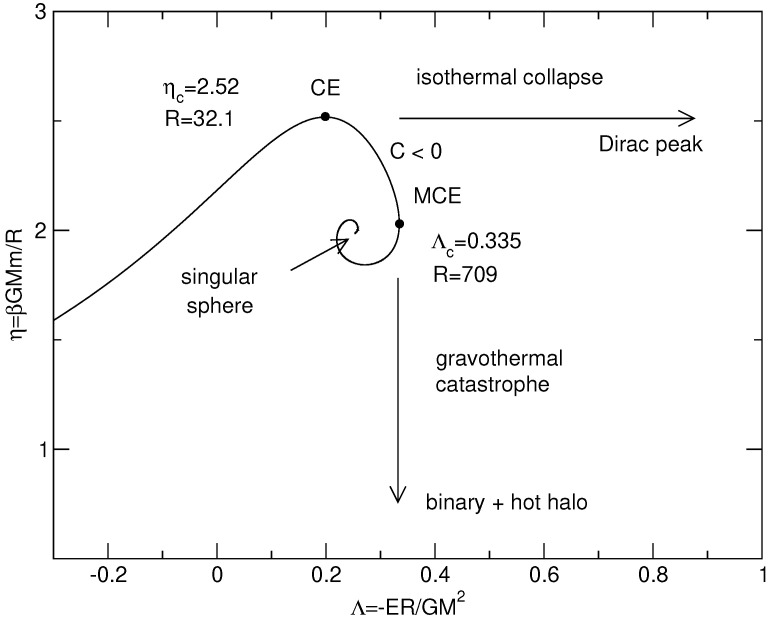
Series of equilibria (caloric curve) for classical self-gravitating systems in a spherical box. There is a region of ensemble inequivalence between points CE and MCE where the specific heat C=dE/dT is negative. For $\Lambda =-ER/GM^{2}>\Lambda_{c}$ in the microcanonical ensemble or for $\eta =\beta GMm/R>\eta_{c}$ in the canonical ensemble, there is no equilibrium state, and the system undergoes a gravitational collapse (gravothermal catastrophe in the microcanonical ensemble and isothermal collapse in the canonical ensemble).

For self-gravitating systems, the Boltzmann entropy has no maximum at fixed mass and energy in an unbounded domain [54]. This is associated with the evaporation of high-energy stars. The Boltzmann entropy does not even have an extremum in an unbounded domain. Indeed, the Boltzmann distribution coupled with the Poisson equation has an infinite mass (the density decreases at large distances as $\rho ∼r^{-2}$) [54]. We shall therefore work in a spherical box of radius *R*. In that case, there can be extrema of Boltzmann entropy at fixed mass and energy. We can compute them and plot the series of equilibria (see, e.g., [54] for technical details). It has the form of a spiral represented in Figure 1. There is no global maximum of entropy at fixed mass and energy [54] in agreement with the divergence of g(E) when a=0, but there can be metastable states (local maxima of entropy at fixed mass and energy) and unstable states (saddle points of entropy at fixed mass and energy). The following results can be proven:

(i) There is no statistical equilibrium state (no entropy extremum) when $E<-0.335GM^{2}/R$.
(ii) Stable equilibrium states exist for $E>-0.335GM^{2}/R$ and R=ρ(0)/ρ(R)<709. They are metastable (local entropy maxima).
(iii) Equilibrium states with $E>-0.335GM^{2}/R$ and R=ρ(0)/ρ(R)>709 are unstable (saddle points of entropy).

These stability results can be established by using the Poincaré turning point criterion [15,55,56]^14^ according to which:

(a) A change in stability in the microcanonical ensemble can occur only at a turning point of energy.
(b) A mode of stability is lost when the caloric curve β(−E) rotates clockwise and gained when it rotates anticlockwise.

These stability results can be confirmed by studying the eigenvalue equation associated with the second variations of entropy [10,54].

The thermodynamics of a system of classical particles in gravitational interaction in a box without small-scale cut-off has been considered in the seminal works of Antonov [57] and Lynden–Bell and Wood [17] in a mean-field approximation. At high energies, the system is in a gaseous phase. Strictly speaking, gaseous states are only *metastable* equilibrium states (local entropy maxima). However, for N≫1, these metastable states are long-lived [29] and represent the observed equilibrium structures in the universe, like globular clusters [47]. Below the critical energy $E_{c}=-0.335GM^{2}/R$ (Antonov energy), the system cannot be in statistical equilibrium, and it undergoes a gravitational collapse (core collapse). This is called *gravothermal catastrophe* [17] in the microcanonical ensemble, where the energy *E* is fixed. It can be interpreted as a zeroth-order phase transition occurring at a spinodal point $E_{c}$. The gravothermal catastrophe ultimately leads to a *binary star* surrounded by a hot halo [10,44,54]. The process of core collapse in the microcanonical ensemble (fixed energy) has been studied dynamically with the aid of fluid equations [58,59,60,61] or with the orbit-averaged Fokker–Planck equation [62,63] (see the conclusion for more details).

## 3. Self-Gravitating Brownian Particles in the Canonical Ensemble

### 3.1. Langevin Equations

We consider a system of *N* Brownian particles in gravitational interaction. This model has been introduced and studied by Chavanis and Sire in a series of papers [22,30,54,64,65,66,67,68,69,70,71,72]. The equations of motion consist in *N* coupled stochastic Langevin equations of the form

(16) $$\begin{array}{c} \frac{dr_{i}}{dt}=v_{i},\;\frac{dv_{i}}{dt}=-\xi v_{i}-\frac{1}{m}\nabla_{i}U\left(r_{1},\ldots ,r_{N}\right)+\sqrt{2D}R_{i}\left(t\right). \end{array}$$

The particles interact via self-gravity and, in addition, they are subject to a friction force and a stochastic force. Here, ξ is the friction coefficient, *D* is the diffusion coefficient in velocity space, and $R_{i}\left(t\right)$ is a Gaussian white noise satisfying $\langle R_{i}\left(t\right)\rangle =0$ and $\langle R_{a,i}\left(t\right)R_{b,j}\left(t^{\prime }\right)\rangle =\delta_{ij}\delta_{ab}\delta \left(t-t^{\prime }\right)$, where a,b=1,2,3 refer to the coordinates of space and i,j=1,…,N to the particles. These additional terms model the coupling of the particles with a thermal bath of non-gravitational origin. We thus have to deal with a dissipative system for which the energy is not conserved. What is fixed instead of the energy is the temperature *T*, which is given by the Einstein relation (see below)

(17) $$\begin{array}{c} T=\frac{Dm}{\xi }. \end{array}$$

Since D∝T, the temperature measures the strength of the stochastic force. As we shall see, the correct statistical ensemble for a system of Brownian particles in interaction is the canonical ensemble, where the control parameter is the temperature *T*.

### 3.2. Canonical Distribution

The evolution of the *N*-body distribution function is governed by the *N*-body Fokker–Planck (Kramers) equation [20,21]

(18) $$\frac{\partial P_{N}}{\partial t}+\sum_{i=1}^{N}\left(v_{i}\cdot \frac{\partial P_{N}}{\partial r_{i}}+F_{i}\cdot \frac{\partial P_{N}}{\partial v_{i}}\right)=\sum_{i=1}^{N}\frac{\partial }{\partial v_{i}}\cdot \left[D\frac{\partial P_{N}}{\partial v_{i}}+\xi P_{N}v_{i}\right].$$

The stationary solution of this equation is the canonical distribution

(19) $$\begin{array}{c} P_{N}\left(r_{1},v_{1},\ldots ,r_{N},v_{N}\right)=\frac{1}{Z(\beta )}e^{-\beta H}, \end{array}$$

where β=1/T is the inverse temperature, provided that the coefficients of diffusion and friction are related to each other by the Einstein relation (17).^15^ We note that the canonical distribution (19) is the only stationary solution of the *N*-body Kramers Equation (18). Furthermore, one can prove an *H*-theorem for this equation in terms of an *N*-body free energy [20,21]. As a result, a system of Brownian particles in interaction relaxes towards the canonical distribution (19) for t→+∞ provided that this distribution exists. The normalization factor of the canonical distribution function

(20) $$Z\left(\beta \right)=\int e^{-\beta H}\prod_{i=1}^{N}dr_{i}dv_{i}$$

is the partition function. This is the quantity of fundamental interest in the canonical ensemble. The free energy is defined by

(21) $$\begin{array}{c} F(T)=-T\ln Z. \end{array}$$

The average energy

(22) $$\begin{array}{c} \langle E\rangle =\int P_{N}H\prod_{i}dr_{i}dv_{i} \end{array}$$

can be written as

(23) $$\begin{array}{c} \langle E\rangle =\frac{1}{Z}\int e^{-\beta H}H\prod_{i}dr_{i}dv_{i}=-\frac{1}{Z}\frac{d}{d\beta }\int e^{-\beta H}\prod_{i}dr_{i}dv_{i}, \end{array}$$

yielding

(24) $$\begin{array}{c} \langle E\rangle =-\frac{1}{Z}\frac{dZ}{d\beta }=-\frac{d\ln Z}{d\beta }=T^{2}\frac{d\ln Z}{dT}. \end{array}$$

The average energy is a function of the temperature. The function 〈E〉(T) defines the canonical caloric curve. Combining Equation (21) with Equation (24), we obtain

(25) $$\begin{array}{c} \langle E\rangle =-T^{2}\frac{d}{dT}\left(\frac{F}{T}\right)=-T\frac{dF}{dT}+F. \end{array}$$

This equation shows that the function F(T)/T attains its minimum value at the temperature at which 〈E〉(T)=0. By the same method as above, we find that

(26) $$\begin{array}{c} \langle E^{2}\rangle =\frac{1}{Z}\frac{d^{2}Z}{d\beta^{2}}. \end{array}$$

Therefore, introducing the fluctuation of energy ΔE=E−〈E〉, we obtain

(27) $$\begin{array}{c} \langle {\left(\Delta E\right)}^{2}\rangle =\langle E^{2}\rangle -{\langle E\rangle }^{2}=\frac{1}{Z}\frac{d^{2}Z}{d\beta^{2}}-\frac{1}{Z^{2}}{\left(\frac{dZ}{d\beta }\right)}^{2}=\frac{d^{2}\ln Z}{d\beta^{2}}. \end{array}$$

Comparing Equations (24) and (27) we arrive at the following relation.^16^

(28) $$\begin{array}{c} C\equiv \frac{d\langle E\rangle }{dT}=\beta^{2}\langle {\left(\Delta E\right)}^{2}\rangle >0, \end{array}$$

where *C* is the canonical specific heat. This relation shows that, in the canonical ensemble, the specific heat *C* is always positive.

**Remark** **3.** *It is easy to see in physical terms why no equilibrium is possible for a system of negative specific heat in contact with a heat bath. If the system is cooler than the bath it will accept heat from it and grow still cooler, becoming unstable. Similarly, if the system is hotter than the bath, it will lose heat and become hotter.*

**Remark** **4.** *The N-body Langevin Equations (16) and the N-body Fokker–Planck Equation (18) are equivalent to a stochastic kinetic equation of the form [76]*

(29) $$\begin{array}{c} \frac{\partial f_{d}}{\partial t}+v\cdot \frac{\partial f_{d}}{\partial r}-\nabla \Phi_{d}\cdot \frac{\partial f_{d}}{\partial v}=\frac{\partial }{\partial v}\cdot \left[D\left(\frac{\partial f_{d}}{\partial v}+\beta mf_{d}v\right)\right]+\frac{\partial }{\partial v}\cdot \left(\sqrt{2Dmf_{d}}\;Q\left(r,v,t\right)\right), \end{array}$$

(30) $$\begin{array}{c} \Phi_{d}\left(r,t\right)=\int u(|r-r^{\prime }\left|\right)f_{d}\left(r^{\prime },v^{\prime },t\right)\;dr^{\prime }dv^{\prime }, \end{array}$$

*for the discrete (exact) distribution function $f_{d}\left(r,v,t\right)=\sum_{i}m\delta \left(r-r_{i}\left(t\right)\right)\delta \left(v-v_{i}\left(t\right)\right)$ made of a sum of N Dirac δ-functions. Here, Q(r,v,t) is a Gaussian white noise satisfying 〈Q(r,v,t)〉=0 and $\langle Q_{\alpha }\left(r,v,t\right)Q_{\beta }\left(r^{\prime },v^{\prime },t^{\prime }\right)\rangle =\delta_{\alpha \beta }\delta \left(r-r^{\prime }\right)\delta \left(v-v^{\prime }\right)\delta \left(t-t^{\prime }\right)$. Equation (29) is a generalization, valid for inertial systems, of the Dean [77] equation. See, e.g., [8] and references therein for additional comments on this type of stochastic kinetic equations.*

### 3.3. Divergences at Short and Large Distances

For self-gravitating systems in d=3 dimensions, if the domain is unlimited, the partition function (20) diverges when the distribution of particles is spread to infinity [10]. Therefore, there is no equilibrium state in a strict sense. A self-gravitating Brownian gas has the tendency to evaporate and disperse to infinity.

When the system is confined within a box, the partition function (20) diverges when all the particles approach each other [10]. Therefore, the strict statistical equilibrium state in the canonical ensemble is a Dirac peak containing the whole mass [78]. However, there may exist other statistical equilibrium states in the form of gaseous configurations where the particles are at large distances from each other. These states correspond to robust metastable equilibrium states with very long lifetimes scaling as $e^{N}\;t_{D}$ [29]. They only exist at sufficiently high temperatures.

In order to regularize the divergence of the partition function at small separations and make the problem be well defined mathematically, we can use a softening potential or consider the case of hard spheres with size *a*.^17^ We note that, contrary to the density of states in the microcanonical ensemble, the partition function already diverges for N=2 when the gravitational potential is not regularized at short distances (i.e., when a=0).

### 3.4. Thermodynamic Limit N→+∞

For systems with long-range interactions, we can define a proper thermodynamic limit N→+∞ with βGMm/R∼1, $ER/GM^{2}∼1$ and a/R∼1 in which the system is extensive but remains fundamentally nonadditive. In this limit, we can make a mean field approximation and develop a thermodynamic approach. Indeed, in the canonical ensemble, we can write the partition function Z(β) under the form

(31) $$\begin{array}{c} Z\left(\beta \right)=\int e^{-\beta N\varphi [f]}\delta \left(M-M\left[f\right]\right)Df, \end{array}$$

where

(32) $$\begin{array}{c} F[f]=N\varphi [f]=E[f]-TS[f] \end{array}$$

is the Helmholtz free energy and the integral in Equation (31) runs over all possible distribution functions f(r,v) that yield the correct mass *M*. It is important to note that for systems with long-range interactions, the free energy is proportional to *N*. As a result, in the thermodynamic limit N→+∞, the integral in Equation (31) is dominated by the macrostate that minimizes the free energy at fixed mass (and temperature). This leads to the thermodynamical principle

(33) $$\begin{array}{c} F\left(T\right)=\min_{f}\left\{F[f]|M\right\}, \end{array}$$

which determines the most probable equilibrium state in the canonical ensemble. This can be viewed as a result of large deviations.

In order for Z(β) to be finite, we must introduce a small-scale regularization. As in Section 2.4, we can either consider the case of classical pointlike particles described by the Boltzmann entropy and use a softening gravitational potential or use the original gravitational potential (without softening) and take into account the size *a* of the particles through, e.g., the Ornstein [52] generalized entropy associated with the van der Waals equation of state [53]. We could also consider the case of fermions and use the Fermi–Dirac entropy in phase space [28] or configuration space [39]. The study of gravitational phase transitions in these different situations, depending on the value of *a*, is discussed in [15,28,39]. Below, to simplify the discussion, we shall consider the limit a→0. Therefore, we use the Boltzmann entropy (15) and the original gravitational potential of interaction $U=-\sum_{i<j}Gm^{2}/\left|r_{i}-r_{j}\right|$. In that case, the density of state Z(β) diverges but the minimization problem (33) may still have a solution corresponding to a long-lived metastable state.

### 3.5. Metastable States

Let us recall well-known results concerning the minimization problem (33) for self-gravitating point masses (a=0).

For self-gravitating systems, the Boltzmann free energy has no minimum at fixed mass in an unbounded domain [40,54]. This is associated with the evaporation of high-energy particles. The Boltzmann free energy has not even an extremum in an unbounded domain. Indeed, the Boltzmann distribution coupled to the Poisson equation has an infinite mass (the density decreases at large distances as $\rho ∼r^{-2}$) [40,54]. We shall therefore work in a spherical box of radius *R*. In that case, there can be extrema of Boltzmann free energy at a fixed mass. We can compute them and plot the series of equilibria (see, e.g., [40,54] for technical details). It has the form of a spiral represented in Figure 1. There is no global minimum of free energy at fixed mass [40,54] in agreement with the divergence of Z(β) when a=0, but there can be metastable states (local minima of free energy at a fixed mass) and unstable states (saddle points of free energy at a fixed mass). The following results can be proven:

(i) There is no statistical equilibrium state (no extremum of free energy) when T<GMm/(2.52R);
(ii) Stable equilibrium states exist for T>GMm/(2.52R) and R=ρ(0)/ρ(R)<32.1. They are metastable (local minima of free energy).
(iii) Equilibrium states with T>GMm/(2.52R) and R=ρ(0)/ρ(R)>32.1 are unstable (saddle points of free energy).

These stability results can be established by using the Poincaré turning point criterion [15,55,56] according to which:

(a) A change in stability in the canonical ensemble can occur only at a turning point of temperature.
(b) A mode of stability is lost when the caloric curve β(−E) rotates clockwise and gained when it rotates anticlockwise.

These stability results can be confirmed by studying the eigenvalue equation associated with the second variations of free energy [40,54].

The thermodynamics of a system of classical particles in gravitational interaction in a box without a small-scale cut-off has been treated in the canonical ensemble by Chavanis [40] by using a mean field approximation. At high temperatures, the system is in a gaseous phase. In a strict sense, gaseous states are only *metastable* equilibrium states (local minima of free energy). However, for N≫1, these metastable states are long-lived [29]. Below the critical temperature $T_{c}=GMm/\left(2.52R\right)$ (Emden [79] temperature), the system cannot be in statistical equilibrium, and it undergoes a gravitational collapse (core collapse). This is called *isothermal collapse* [40] in the canonical ensemble where the temperature *T* is fixed. It can be interpreted as a zeroth-order phase transition occurring at a spinodal point $T_{c}$. The isothermal collapse ultimately leads to a *Dirac peak* containing the whole mass [40,54,78]. The process of core collapse in the canonical ensemble (fixed temperature) has been studied dynamically with the aid of the Smoluchowski–Poisson equations describing a gas of self-gravitating Brownian particles in the strong friction limit ξ→+∞ [22,54]. The pre-collapse is self-similar, and the density profile develops a finite time singularity (core collapse). The central density $\rho_{0}∼{\left(t_{\mathrm{coll}}-t\right)}^{-1}$ becomes infinite in a finite time $t_{\mathrm{coll}}$ and the core radius $r_{0}∼{\left(t_{\mathrm{coll}}-t\right)}^{1/2}$ tends to zero, leading to a singular density profile $\rho ∼r^{-2}$. We note, however, that the core mass $M_{0}\left(t\right)∼{\left(t_{\mathrm{coll}}-t\right)}^{1/2}$ tends to zero at the collapse time. Therefore, “the central singularity contains no mass” in apparent contradiction with the thermodynamical expectation. In fact, the collapse continues after the singularity and a Dirac peak containing all the mass is finally formed in the post-collapse regime [64].

### 3.6. Ensemble Inequivalence

By comparing the results of Section 2.4 and Section 3.4, we see that the series of equilibria displays a region of ensemble inequivalence between points CE and MCE in Figure 1. The equilibrium states in this region are stable in the microcanonical ensemble, while they are unstable in the canonical ensemble. They have a negative specific heat, which is allowed in the microcanonical ensemble but forbidden in the canonical ensemble. We also note that the equilibrium states past point MCE are unstable in both the canonical and microcanonical ensembles, whatever the sign of the specific heat. In the canonical ensemble, stability is lost at point CE when the specific heat passes from positive to negative. In the microcanonical ensemble, stability is lost at point MCE when the specific heat passes from negative to positive. The strict caloric curve is obtained by keeping only fully stable states. In the present case, there are no fully stable states, so there is no caloric curve in a strict sense. This is associated with the divergence of g(E) and Z(β) when a=0. The physical caloric curve is obtained by keeping fully stable and metastable states (in the present case only metastable states) and discarding unstable states. In the canonical ensemble, the physical caloric curve stops at point CE. For $T<T_{c}$, the system undergoes an isothermal collapse. In the microcanonical ensemble, the physical caloric curve stops at point MCE. For $E<E_{c}$, the system undergoes a gravothermal catastrophe. The generalization of these results when a small-scale cut-off *a* is introduced, and the description of the nature of gravitational phase transitions as a function of *a*, are treated in [15].

## 4. Fluctuations of Energy in the Canonical Ensemble

In this section, we discuss the fluctuations of energy in the canonical ensemble.

### 4.1. Distribution of Energies and Thermodynamic Potential

In the canonical ensemble, the distribution of energies at temperature *T* is given by

(34) $$\begin{array}{c} P\left(E\right)=\frac{1}{Z(T)}g\left(E\right)e^{-\beta E}\;\mathrm{with}\;Z\left(T\right)=\int g\left(E\right)e^{-\beta E}\;dE. \end{array}$$

This distribution can be obtained by integrating the Gibbs distribution from Equation (19) over the microstates that have the energy *E* with the density of states g(E). Introducing the entropy from Equation (5), we can rewrite this distribution as

(35) $$\begin{array}{c} P\left(E\right)=\frac{1}{Z(T)}e^{-\beta F(E)}\;\mathrm{with}\;Z\left(T\right)=\int e^{-\beta F(E)}\;dE, \end{array}$$

where we have defined the free energy

(36) $$\begin{array}{c} F(E)=E-TS(E). \end{array}$$

This is a function of the energy *E* for a given temperature *T*. It plays the role of a thermodynamic potential. It should not be confused with the free energy F(T) from Equation (21), which is a function of *T* alone. From Equation (36), we have

(37) $$\begin{array}{c} F^{\prime }\left(E\right)=1-TS^{\prime }\left(E\right) \end{array}$$

and

(38) $$\begin{array}{c} F^{\prime \prime }\left(E\right)=-TS^{\prime \prime }\left(E\right). \end{array}$$

### 4.2. Equilibrium States

The equilibrium states at a given temperature *T* are determined by the condition $F^{\prime }\left(E_{\mathrm{eq}}\right)=0$ giving

(39) $$\begin{array}{c} \frac{1}{T}=S^{\prime }\left(E_{\mathrm{eq}}\right). \end{array}$$

Accordingly, the energies $E_{\mathrm{eq}}\left(T\right)$ of the equilibrium states at fixed temperature *T* can be obtained from the microcanonical caloric curve T(E) [see Equation (6) in Section 2] by rotating this curve by $90^{o}$. In other words, $E_{\mathrm{eq}}\left(T\right)$ is the inverse of the function T(E). When T(E) is non-monotonic (displaying a region of negative specific heats) like in the case of self-gravitating systems, the function $E_{\mathrm{eq}}\left(T\right)$ is multi-valued. We can have stable (global minimum of *F*), metastable (local minimum of *F*), and unstable (maximum of *F*) equilibrium states. The global minimum of F(E) determines the most probable energy at temperature *T*. The second variations of the free energy at an equilibrium state are given by

(40) $$\begin{array}{c} F^{\prime \prime }\left(E_{\mathrm{eq}}\right)=-TS^{\prime \prime }\left(E_{\mathrm{eq}}\right). \end{array}$$

Applying Equation (8) at $E=E_{\mathrm{eq}}$ where $T(E_{\mathrm{eq}})=T$, we can rewrite Equation (40) as

(41) $$\begin{array}{c} F^{\prime \prime }\left(E_{\mathrm{eq}}\right)=\frac{1}{TC(E_{\mathrm{eq}})}, \end{array}$$

where C(E)=dE/dT is the microcanonical specific heat. Therefore,

(42) $$\begin{array}{c} F^{\prime \prime }\left(E_{S}\right)=\frac{1}{TC_{S}}>0,\;F^{\prime \prime }\left(E_{M}\right)=\frac{1}{TC_{M}}>0,\;F^{\prime \prime }\left(E_{U}\right)=\frac{1}{TC_{U}}<0 \end{array}$$

for the stable (S), metastable (M), and unstable (U) equilibrium states, respectively. From Equation (41), we see that a minimum of free energy has a positive specific heat, while a maximum of free energy has a negative specific heat. We will see in Section 5, Section 6 and Section 7 that minima of free energy F(E) are dynamically stable in the canonical ensemble while maxima are unstable.

### 4.3. Mean Field Approximation and Metastable States

If the integral in Equation (35) is dominated by the global minimum of F(E) denoted $E_{S}\left(T\right)$, we can make the mean field approximation

(43) $$\begin{array}{c} Z\left(T\right)∼e^{-\beta F[E_{S}\left(T\right)]}. \end{array}$$

As a result, the free energy (21) can be written as

(44) $$\begin{array}{c} F\left(T\right)≃F\left[E_{S}\left(T\right)\right]=E_{S}\left(T\right)-TS\left[E_{S}\left(T\right)\right]. \end{array}$$

According to Equation (25), the average energy is given by

(45) $$\begin{array}{c} \langle E\rangle \left(T\right)≃-T\left(E_{S}^{\prime }\left(T\right)-S\left[E_{S}\left(T\right)\right]-TS^{\prime }\left[E_{S}\left(T\right)\right]E_{S}^{\prime }\left(T\right)\right)+E_{S}\left(T\right)-TS\left[E_{S}\left(T\right)\right]. \end{array}$$

Using Equation (6), we can simplify the term in parenthesis and obtain

(46) $$\begin{array}{c} \langle E\rangle \left(T\right)≃E_{S}\left(T\right). \end{array}$$

This determines the caloric curve in the canonical ensemble in the mean field approximation. For systems with long-range interactions, the mean field approximation is exact in a proper thermodynamic limit N→+∞. However, the Formula (46) is not exact for small values of *N* (e.g., for N=2). In addition, even in the thermodynamic limit N→+∞, this formula misses the important notion of metastable states. It is therefore necessary to introduce different notions of caloric curves.

In order to take into account all (stable, metastable and unstable) equilibrium states from Equation (39), we can define an energy $E_{\mathrm{eq}}\left(T\right)$, which is the inverse function of T(E) defined by Equation (6) in the microcanonical ensemble. The corresponding free energy is

(47) $$\begin{array}{c} F_{\mathrm{eq}}\left(T\right)=F\left[E_{\mathrm{eq}}\left(T\right)\right]=E_{\mathrm{eq}}\left(T\right)-TS\left[E_{\mathrm{eq}}\left(T\right)\right]. \end{array}$$

The functions $E_{\mathrm{eq}}\left(T\right)$ and $F_{\mathrm{eq}}\left(T\right)$ may be multi-valued, unlike the functions 〈E〉(T) and F(T). The function $F_{\mathrm{eq}}\left(T\right)$ can be written in parametric form as

(48) $$\begin{array}{c} \left\{ \begin{array}{c} F_{\mathrm{eq}}=E-T\left(E\right)S\left(E\right),\\ T=T(E), \end{array}\right. \end{array}$$

with running parameter *E*. The curves $E_{\mathrm{eq}}\left(T\right)$ and $F_{\mathrm{eq}}\left(T\right)$, displaying stable, metastable and unstable states, form the series of equilibria. The metastable and unstable branches merge at a critical point where the temperature $T_{c}$ is minimum. There can also be a critical point where the temperature $T_{*}$ is maximum. These two points are called spinodal points (see [15] and Section 3 and Section 11 of the present paper).

From the series of equilibria $E_{\mathrm{eq}}\left(T\right)$ and $F_{\mathrm{eq}}\left(T\right)$, we can define the physical caloric curve $E_{\mathrm{phys}}\left(T\right)$ and the physical free energy $F_{\mathrm{phys}}\left(T\right)$ that contain only stable and metastable equilibrium states. Finally, we can define the strict caloric curve $E_{\mathrm{strict}}\left(T\right)$ and the strict free energy $F_{\mathrm{strict}}\left(T\right)$ that contain only stable equilibrium states. The strict caloric curve $E_{\mathrm{strict}}\left(T\right)$ and the strict free energy $F_{\mathrm{strict}}\left(T\right)$ coincide with the exact caloric curve 〈E〉(T) and the exact free energy F(T) in the thermodynamical limit N→+∞, but they may deviate from them for smaller values of *N*. In conclusion, we have to distinguish the exact caloric curve 〈E〉(T) defined by Equation (24), the strict caloric curve $E_{\mathrm{strict}}\left(T\right)$ that contains only stable equilibrium states, the physical caloric curve $E_{\mathrm{phys}}\left(T\right)$ that contains stable and metastable equilibrium states, and the series of equilibria $E_{\mathrm{eq}}\left(T\right)$ that contains all (stable, metastable and unstable) equilibrium states.

**Remark** **5.** *We note that the series of equilibria in the canonical ensemble coincides with the microcanonical caloric curve. The physical caloric curve in the canonical ensemble differs from the microcanonical caloric curve by the removal of canonically unstable states. The strict caloric curve in the canonical ensemble is in general close to the exact canonical caloric curve (they coincide for N→+∞). They differ from the microcanonical caloric curve in the sense that the region of negative specific heat in the microcanonical ensemble is replaced by a first-order phase transition in the canonical ensemble. An illustration of these results is given in Ref. [15] for self-gravitating fermions (N→+∞) and in Section 11 of the present paper for the binary star model (N=2).*

### 4.4. Gaussian Distribution of Fluctuations

Close to equilibrium, we can expand the free energy F(E) up to the second order. Using $F^{\prime }\left(E_{\mathrm{eq}}\right)=0$, we obtain $F\left(E\right)=F\left(E_{\mathrm{eq}}\right)+\frac{1}{2}F^{\prime \prime }\left(E_{\mathrm{eq}}\right){\left(E-E_{\mathrm{eq}}\right)}^{2}+\ldots$. Then, Equation (35) leads to the Gaussian distribution of fluctuations

(49) $$\begin{array}{c} P\left(\Delta E\right)=\frac{1}{Z(T)}e^{-\frac{1}{2}\beta F^{\prime \prime }\left(E_{\mathrm{eq}}\right){\left(\Delta E\right)}^{2}}. \end{array}$$

Using Equation (38), we can write this distribution as

(50) $$\begin{array}{c} P\left(\Delta E\right)=\frac{1}{Z(T)}e^{\frac{1}{2}S^{\prime \prime }\left(E_{\mathrm{eq}}\right){\left(\Delta E\right)}^{2}}. \end{array}$$

Finally, using Equation (8) or Equation (41), we obtain

(51) $$\begin{array}{c} P\left(\Delta E\right)=\frac{1}{Z(T)}e^{-\frac{{\left(\Delta E\right)}^{2}}{2CT^{2}}}. \end{array}$$

The prefactor (the inverse of the partition function) in these equations can be obtained from the normalization condition $\int_{-\infty }^{+\infty }P\left(\Delta E\right)\;d\left(\Delta E\right)=1$. We can calculate the variance of the fluctuations of energy directly from Equation (51) and obtain the result

(52) $$\begin{array}{c} \langle {\left(\Delta E\right)}^{2}\rangle =CT^{2}. \end{array}$$

As expected, it coincides with the Gibbs–Einstein formula from Equation (28).

## 5. Lifetime of Metastable States

In this section, we determine the lifetime of metastable states by using an adaptation of the Kramers formula.

### 5.1. Fokker–Planck Equation in the Weak Friction Limit

We consider a system of Brownian particles with long-range interactions in the canonical ensemble. In the weak friction limit ξ→0, the *N*-body distribution function $P_{N}\left(r_{1},v_{1},\ldots ,r_{N},v_{N},t\right)$ is a function $P_{N}\left(E,t\right)$ of the energy *E* alone, and its time evolution can be shown to be governed by a Fokker–Planck equation of the form [29]

(53) $$\begin{array}{c} g\left(E\right)\frac{\partial P_{N}}{\partial t}\left(E,t\right)=3MD\frac{\partial }{\partial E}\left[I\left(E\right)\left(\frac{\partial P_{N}}{\partial E}+\beta P_{N}\right)\right], \end{array}$$

where

(54) $$\begin{array}{c} I\left(E\right)=\int_{H\leq E}\prod_{i=1}^{N}dr_{i}dv_{i} \end{array}$$

is the hypervolume of phase space^18^ with energy less than *E* (action) and

(55) $$\begin{array}{c} g\left(E\right)=\frac{dI}{dE}=\int \delta \left(E-H\right)\prod_{i=1}^{N}dr_{i}dv_{i} \end{array}$$

is the density of states already introduced in Equation (4). The quantity g(E)dE represents the phase-space hypervolume with energy between *E* and E+dE. The condition H≤E is equivalent to $\frac{1}{2}\sum_{i=1}^{N}mv_{i}^{2}=E-U\left(r_{1},\ldots ,r_{N}\right)$. If we define $x_{i}=\sqrt{m/2}\;v_{i}$ for i=1,…,N, the condition H≤E can be rewritten as $\sum_{i=1}^{N}x_{i}^{2}\leq E-U$. It characterizes a 3N-dimensional sphere of “radius” $\sqrt{E-U}$. Integrating over the velocities, we can rewrite Equation (54) as

(56) $$\begin{array}{c} I\left(E\right)={\left(\frac{2}{m}\right)}^{3N/2}V_{3N}\int {\left(E-U\right)}^{3N/2}\prod_{i=1}^{N}dr_{i}, \end{array}$$

where $V_{d}=2\pi^{d/2}/\left[d\;\Gamma \left(d/2\right)\right]$ is the volume of a hypersphere of unit radius in *d* dimensions. The density of state is then given by

(57) $$\begin{array}{c} g\left(E\right)=\frac{3N}{2}{\left(\frac{2}{m}\right)}^{3N/2}V_{3N}\int {\left(E-U\right)}^{3N/2-1}\prod_{i=1}^{N}dr_{i}. \end{array}$$

The Fokker–Planck Equation (53) relaxes towards the Boltzmann–Gibbs distribution

(58) $$\begin{array}{c} P_{N}^{\mathrm{eq}}\left(E\right)=\frac{1}{Z(\beta )}e^{-\beta E}, \end{array}$$

in agreement with Equation (19).

It is convenient to introduce the distribution of energies defined by $P\left(E,t\right)=P_{N}\left(E,t\right)g\left(E\right)$. Using Equation (53), its evolution in the weak friction limit ξ→0 is governed by the Fokker–Planck equation [29]

(59) $$\begin{array}{c} \frac{\partial P}{\partial t}=\frac{\partial }{\partial E}\left[D\left(E\right)\left(\frac{\partial P}{\partial E}+\beta PF^{\prime }\left(E\right)\right)\right], \end{array}$$

where F(E) is the free energy defined by Equation (36) and D(E) is the the diffusion coefficient in energy space given by

(60) $$\begin{array}{c} D\left(E\right)=3MD\frac{I(E)}{g(E)}. \end{array}$$

We note that the Fokker–Planck Equation (59) has the form of a Smoluchowski equation where the energy *E* plays the role of the position *x* and the free energy F(E) plays the role of the potential V(x). It relaxes towards the Boltzmann distribution

(61) $$\begin{array}{c} P_{\mathrm{eq}}\left(E\right)=\frac{1}{Z(\beta )}e^{-\beta F(E)}, \end{array}$$

in agreement with Equation (35). Using Equation (36), the drift term in the Fokker–Planck Equation (59) can be written as $F_{\mathrm{friction}}=-D\left(E\right)\beta F^{\prime }\left(E\right)=-D\left(E\right)\left(\beta -\beta \left(E\right)\right)$, where β=1/T is the inverse temperature of the bath and $\beta \left(E\right)=S^{\prime }\left(E\right)$ is the microcanonical inverse temperature.

**Remark** **6.** *We can rewrite the Fokker–Planck Equation (53) in terms of the action I as*

(62) $$\begin{array}{c} \frac{\partial P_{N}}{\partial t}\left(I,t\right)=3MD\frac{\partial }{\partial I}\left[\frac{I}{\Omega (I)}\left(\frac{\partial P_{N}}{\partial I}+\beta P_{N}\Omega \right)\right], \end{array}$$

*where we have introduced the pulsation*

(63) $$\begin{array}{c} \Omega \left(I\right)=\frac{dE}{dI}=\frac{1}{g(E)}. \end{array}$$

*The diffusion coefficient in action space is $D_{K}\left(I\right)=3MDI/\Omega \left(I\right)$. This is similar to the original results of Kramers [80].*

### 5.2. Kramers Formula

For a system described by a Fokker–Planck of the form of Equation (59), the lifetime of a metastable state (local minimum of the potential F(E)) is given by the Kramers formula (see Appendix E)

(64) $$\begin{array}{c} t_{\mathrm{life}}=\frac{\pi }{D\left(E_{U}\right)\beta \omega_{M}\left|\omega_{U}\right|}e^{\beta \Delta F}, \end{array}$$

where $\Delta F=F\left(E_{U}\right)-F\left(E_{M}\right)$ is the barrier of free energy between the metastable state $E_{M}$ and the unstable state $E_{U}$, and we have introduced the squared pulsations (see Section 7.2)

(65) $$\begin{array}{c} \omega_{M}^{2}=F^{\prime \prime }\left(E_{M}\right)>0,\;\omega_{U}^{2}=F^{\prime \prime }\left(E_{U}\right)<0. \end{array}$$

Using Equation (41), we have the relations

(66) $$\begin{array}{c} \omega_{M}^{2}=\frac{1}{TC(E_{M})}>0,\;\omega_{U}^{2}=\frac{1}{TC(E_{U})}<0, \end{array}$$

where $C(E_{M})>0$ and $C(E_{U})<0$ are the specific heats of the metastable and unstable states, respectively. We note that the general relation

(67) $$\begin{array}{c} \omega^{2}=F^{\prime \prime }\left(E_{\mathrm{eq}}\right)=\frac{1}{TC(E_{\mathrm{eq}})} \end{array}$$

can be written as

(68) $$\begin{array}{c} \omega^{2}=\frac{1}{T}\frac{dT}{dE}. \end{array}$$

This equation relates the stability of the equilibrium states in the canonical ensemble ($\omega^{2}>0$ for stable states and $\omega^{2}<0$ for unstable states) to the slope of the caloric curve (C=dE/dT>0 for stable states and C=dE/dT<0 for unstable states). The change of stability in the canonical ensemble ($\omega^{2}=0$) occurs at a turning point of temperature $C^{-1}=dT/dE=0$). This is a particular form of the Poincaré [56] turning point criterion. Using Equation (66), the Kramers Formula (64) can be rewritten as [29]:

(69) $$\begin{array}{c} t_{\mathrm{life}}=\pi T^{2}\frac{\sqrt{C_{M}\left|C_{U}\right|}}{D_{U}}e^{\Delta F/T}. \end{array}$$

This is a very general result giving the lifetime of a metastable state $E_{M}$ (local minimum of F(E)). This formula also applies to the stable state $E_{S}$ (global minimum of F(E)), which is also metastable when a local minimum of free energy exists and *N* is small. Using Equation (65), we have, equivalently,

(70) $$\begin{array}{c} t_{\mathrm{life}}=\frac{\pi }{D_{U}\beta \sqrt{F^{\prime \prime }\left(E_{M}\right)\left|F^{\prime \prime }\left(E_{U}\right)\right|}}e^{\beta \Delta F}. \end{array}$$

**Remark** **7.** *A more accurate expression of the lifetime of a metastable state (see Appendix E) is given by [29]*

(71) $$\begin{array}{c} t_{\mathrm{life}}=\int_{E_{M}}^{E_{U}}dE\;\frac{e^{\beta F(E)}}{D(E)}\int_{E_{\min}}^{E}dE^{\prime }e^{-\beta F(E^{\prime })}. \end{array}$$

*This formula is valid when $E_{M}<E_{U}$. The case $E_{M}>E_{U}$ can be obtained similarly, leading to*

(72) $$\begin{array}{c} t_{\mathrm{life}}=\int_{E_{U}}^{E_{M}}dE\;\frac{e^{\beta F(E)}}{D(E)}\int_{E}^{+\infty }dE^{\prime }e^{-\beta F(E^{\prime })}. \end{array}$$

## 6. Normal Form of the Thermodynamic Potential Close to the Critical Temperature

Formula (69), giving the lifetime of a metastable state, can be simplified close to the critical point by using the normal (saddle-node) form of the thermodynamic potential.

### 6.1. Saddle Node Form

The series of equilibria $\beta (E_{\mathrm{eq}})$ is determined by Equation (39). Close to the critical point $T_{c}$ (minimum temperature), where $\beta^{\prime }\left(E_{0}\right)=0$, we can make the expansion (up to second order):

(73) $$\begin{array}{c} \beta =\beta_{c}+\frac{1}{2}\beta^{\prime \prime }\left(E_{0}\right){\left(E_{\mathrm{eq}}-E_{0}\right)}^{2}+\ldots \end{array}$$

On the other hand, expanding the thermodynamic potential F(E) defined by Equation (36) close to $E_{0}$, we obtain (up to third order):

(74) $$\begin{array}{c} F\left(E\right)=E_{0}-TS\left(E_{0}\right)+\left[1-TS^{\prime }\left(E_{0}\right)\right]\left(E-E_{0}\right)-\frac{1}{2}TS^{\prime \prime }\left(E_{0}\right){\left(E-E_{0}\right)}^{2}\\ -\frac{1}{6}TS^{\prime \prime \prime }\left(E_{0}\right){\left(E-E_{0}\right)}^{3}+\ldots \end{array}$$

Using Equation (39), we find that

(75) $$\begin{array}{c} S^{\prime }\left(E_{0}\right)=\beta_{c},\;S^{\prime \prime }\left(E_{0}\right)=\beta^{\prime }\left(E_{0}\right)=0,\;S^{\prime \prime \prime }\left(E_{0}\right)=\beta^{\prime \prime }\left(E_{0}\right). \end{array}$$

Therefore, we can rewrite Equation (74) as

(76) $$\begin{array}{c} F\left(E\right)=E_{0}-TS\left(E_{0}\right)+\left(1-\frac{\beta_{c}}{\beta }\right)\left(E-E_{0}\right)-\frac{1}{6}\frac{\beta^{\prime \prime }\left(E_{0}\right)}{\beta }{\left(E-E_{0}\right)}^{3}. \end{array}$$

Since $\beta ≃\beta_{c}$, we can make the additional approximation

(77) $$\begin{array}{c} F\left(E\right)=F_{0}-\frac{\beta_{c}-\beta }{\beta_{c}}\left(E-E_{0}\right)-\frac{1}{6}\frac{\beta^{\prime \prime }\left(E_{0}\right)}{\beta_{c}}{\left(E-E_{0}\right)}^{3}, \end{array}$$

where $F_{0}=E_{0}-T_{c}S\left(E_{0}\right)$. We can check that the condition $F^{\prime }\left(E_{\mathrm{eq}}\right)=0$ returns Equation (73). Equation (73) can be inverted to give

(78) $$\begin{array}{c} E_{\mathrm{eq}}=E_{0}\pm \sqrt{\frac{2}{-\beta^{\prime \prime }\left(E_{0}\right)}}{\left(\beta_{c}-\beta \right)}^{1/2}. \end{array}$$

Using Equations (77) and (78), we find that the barrier of free energy between the metastable state and the unstable state is given by

(79) $$\begin{array}{c} \Delta F=\frac{4}{3\beta_{c}}\sqrt{\frac{2}{-\beta^{\prime \prime }\left(E_{0}\right)}}{\left(\beta_{c}-\beta \right)}^{3/2}. \end{array}$$

The specific heat can be written as

(80) $$\begin{array}{c} C=\frac{dE}{dT}=-\beta^{2}\frac{dE}{d\beta }. \end{array}$$

Using Equation (78), we obtain close to the critical point

(81) $$\begin{array}{c} C=\pm \frac{\beta_{c}^{2}}{2}\sqrt{\frac{2}{-\beta^{\prime \prime }\left(E_{0}\right)}}{\left(\beta_{c}-\beta \right)}^{-1/2} \end{array}$$

or, equivalently,

(82) $$\begin{array}{c} C=\frac{\beta_{c}^{2}}{-\beta^{\prime \prime }\left(E_{0}\right)}{\left(E_{\mathrm{eq}}-E_{0}\right)}^{-1}. \end{array}$$

These equations can also be directly derived from the relation [see Equation (41)]

(83) $$\begin{array}{c} F^{\prime \prime }\left(E_{\mathrm{eq}}\right)=\frac{\beta }{C} \end{array}$$

by using Equation (77).

### 6.2. Lifetime of Metastable States Close to the Critical Point

Close to the critical point, Equation (69) can be rewritten in sufficient approximation as

(84) $$\begin{array}{c} t_{\mathrm{life}}∼\frac{\pi \sqrt{C_{M}\left|C_{U}\right|}}{D(E_{0})\beta_{c}^{2}}e^{\beta_{c}\Delta F}. \end{array}$$

Substituting Equations (79) and (81) into Equation (84), we find that the lifetime of a metastable state close to the critical point is given by

(85) $$\begin{array}{c} t_{\mathrm{life}}∼\frac{\pi }{2D(E_{0})}\sqrt{\frac{2}{-\beta^{\prime \prime }\left(E_{0}\right)}}{\left(\beta_{c}-\beta \right)}^{-1/2}e^{\frac{4}{3}\sqrt{\frac{2}{-\beta^{\prime \prime }\left(E_{0}\right)}}{\left(\beta_{c}-\beta \right)}^{3/2}} \end{array}$$

with $D\left(E_{0}\right)∼3MDE_{0}$. The lifetime of a metastable state is dominated by the exponential term. We can estimate the effective temperature of collapse $\beta_{l}$ due to finite *N* effects by writing

(86) $$\begin{array}{c} \frac{4}{3}\sqrt{\frac{2}{-\beta^{\prime \prime }\left(E_{0}\right)}}{\left(\beta_{c}-\beta_{l}\right)}^{3/2}∼1. \end{array}$$

This gives

(87) $$\begin{array}{c} \beta_{l}=\beta_{c}\left[1-{\left(\frac{3}{4}\right)}^{2/3}\frac{1}{\beta_{c}}{\left(\frac{-\beta^{\prime \prime }\left(E_{0}\right)}{2}\right)}^{1/3}\right]. \end{array}$$

**Remark** **8.** *The Kramers formula assumes ΔF/T≫1 so the above equations for $t_{\mathrm{life}}$ are not valid if we are too close to the collapse temperature $T_{c}$.*

### 6.3. Normalized Variables

Introducing the dimensionless variables of Appendix C, the lifetime of a metastable state given by Equation (69) can be written as

(88) $$\begin{array}{c} t_{\mathrm{life}}=\frac{\pi }{3}\frac{\sqrt{C_{M}\left|C_{U}\right|}}{D_{U}}\frac{t_{K}}{N\eta }e^{N\eta \Delta F}. \end{array}$$

On the other hand, Equations (79) and (81), which are valid close to the critical point, can be written as

(89) $$\begin{array}{c} \Delta F=\frac{4}{3\eta_{c}}\sqrt{\frac{2}{-\eta^{\prime \prime }\left(\Lambda_{0}\right)}}{\left(\eta_{c}-\eta \right)}^{3/2} \end{array}$$

and

(90) $$\begin{array}{c} C=\pm \frac{\eta_{c}^{2}}{2}\sqrt{\frac{2}{-\eta^{\prime \prime }\left(\Lambda_{0}\right)}}{\left(\eta_{c}-\eta \right)}^{-1/2}. \end{array}$$

Substituting Equations (89) and (90) into Equation (88), we find that the lifetime of a metastable state close to the critical point is given by

(91) $$\begin{array}{c} t_{\mathrm{life}}=\frac{\pi }{3}\frac{\eta_{c}}{\sqrt{-2\eta^{\prime \prime }\left(\Lambda_{0}\right)}}\frac{1}{D(\Lambda_{0})}{\left(\eta_{c}-\eta \right)}^{-1/2}e^{\frac{4}{3}N\sqrt{\frac{2}{-\eta^{\prime \prime }\left(\Lambda_{0}\right)}}{\left(\eta_{c}-\eta \right)}^{3/2}}\frac{t_{K}}{N} \end{array}$$

with $D\left(\Lambda_{0}\right)∼-\Lambda_{0}$. The effective temperature of collapse $\eta_{l}$ due to finite *N* effects is

(92) $$\begin{array}{c} \eta_{l}=\eta_{c}\left[1-{\left(\frac{3}{4}\right)}^{2/3}\frac{1}{\eta_{c}}{\left(\frac{-\eta^{\prime \prime }\left(\Lambda_{0}\right)}{2}\right)}^{1/3}N^{-2/3}\right]. \end{array}$$

These relations are general. Below, we compute $\eta_{c}$ and $\eta^{\prime \prime }\left(\Lambda_{0}\right)$ explicitly for N→+∞ (see Section 6.4) and for N=2 (see Section 12).

### 6.4. Large N Systems with a=0

**Figure 2:**
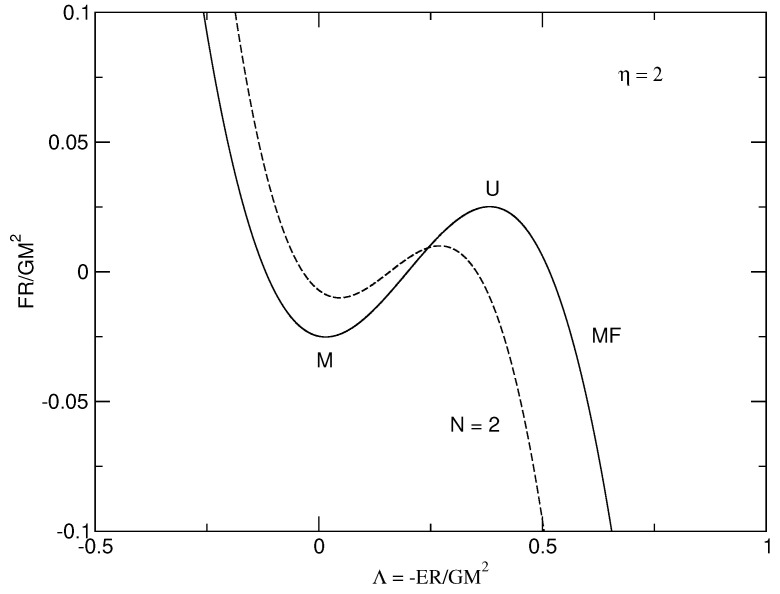
Free energy as a function of the energy at a fixed temperature (here η=2) showing the metastable state, the unstable state, and the barrier of potential. The solid line corresponds to the mean field approximation N→+∞ treated in Section 3. The dashed line corresponds to the case of N=2 particles treated in Section 12. These curves are valid for point-like particles (a=0) close to the critical temperature.

Let us apply the foregoing results to a classical isothermal self-gravitating gas of point-like particles (a=0) enclosed within a box of radius *R* in the mean field limit N→+∞. The series of equilibria (caloric curve) is represented in Figure 1 and the free energy F(E) at a fixed temperature *T* is represented in Figure 2. Using the results of [29], we have

(93) $$\begin{array}{c} \alpha_{c}=8.993195\ldots ,\;\eta_{c}=2.517551\ldots ,\;\Lambda_{0}=\frac{1}{2\eta_{c}}=0.1986057\ldots , \end{array}$$

(94) $$\begin{array}{c} -\eta^{\prime \prime }\left(\Lambda_{0}\right)=\frac{\eta_{c}^{3}}{\eta_{c}-2}=30.8306. \end{array}$$

With these expressions, the lifetime of a metastable state close to the critical point $\eta_{c}$ is explicitly given by

(95) $$\begin{array}{c} t_{\mathrm{life}}=0.3357395\;\frac{1}{D(\Lambda_{0})}{\left(\eta_{c}-\eta \right)}^{-1/2}e^{0.339596\;N{\left(\eta_{c}-\eta \right)}^{3/2}}\frac{t_{K}}{N}. \end{array}$$

We see that the lifetime of a metastable state typically scales as $e^{N}$. Therefore, metastable states are very robust when N≫1. Close to the critical point, there is an attenuation factor scaling as ${\left(\eta_{c}-\eta \right)}^{3/2}$. This scaling was given in [29]. The prefactor in front of the exponential term scales as ${\left(\eta_{c}-\eta \right)}^{-1/2}$. This scaling was not given in [29]. On the other hand, the effective temperature of collapse is explicitly given by

(96) $$\begin{array}{c} \eta_{l}=2.517551\left(1-0.816043\;N^{-2/3}\right). \end{array}$$

This returns the scaling $N^{-2/3}$ obtained in [29]. These scalings are “universal” because they are based on the normal form of the potential close to a critical point. Using the results of [29], we can also show that the density contrast R=ρ(0)/ρ(R) of the equilibrium states close to the critical temperature is given by (see Equations (72) and (102) of [29])

(97) $$\begin{array}{c} R=R_{c}\left[1-\sqrt{\frac{2\eta_{c}}{\eta_{c}-2}}{\left(\eta_{c}-\eta \right)}^{1/2}\right] \end{array}$$

with $R_{c}=\alpha_{c}^{2}/\eta_{c}=32.1255\ldots$. Combined with Equation (92), we find that the effective density contrast of collapse is

(98) $$\begin{array}{c} R_{l}=R_{c}\left[1-{\left(\frac{3\eta_{c}}{8}\right)}^{1/3}{\left(\frac{2\eta_{c}}{\eta_{c}-2}\right)}^{2/3}N^{-1/3}\right]. \end{array}$$

Numerically,

(99) $$\begin{array}{c} R_{l}=32.1255\;\left(1-4.47068\;N^{-1/3}\right). \end{array}$$

This returns the $N^{-1/3}$ scaling obtained in [29] (the prefactor is different because the effective density contrast is defined and calculated in a different manner).

## 7. Dynamical Evolution of the Energy

### 7.1. Langevin Equation

The stochastic Langevin equation for the energy associated with the Fokker–Planck Equation (59) reads

(100) $$\begin{array}{c} \frac{dE}{dt}=-D_{0}\beta \frac{dF}{dE}+\sqrt{2D_{0}}\eta \left(t\right), \end{array}$$

where η(t) is Gaussian white noise satisfying 〈η(t)〉=0 and $\langle \eta \left(t\right)\eta \left(t^{\prime }\right)\rangle =\delta \left(t-t^{\prime }\right)$ (we have replaced D(E) by $D_{0}=D\left(E_{0}\right)$ to simplify the problem, which is a reasonable approximation if we are close to the critical point). This is similar to the equation of motion for an overdamped Brownian particle of unit mass with “position” *E* evolving in a potential F(E). If we neglect the fluctuations, we obtain the deterministic equation

(101) $$\begin{array}{c} \frac{dE}{dt}=-D_{0}\beta F^{\prime }\left(E\right). \end{array}$$

The formal solution of this equation is

(102) $$\begin{array}{c} \int^{E}\frac{dx}{F^{\prime }\left(x\right)}=-D_{0}\beta t. \end{array}$$

Close to the critical temperature $T_{c}$, using the normal form of the potential from Equation (77), the foregoing equations become

(103) $$\begin{array}{c} \frac{dE}{dt}=\frac{1}{2}D_{0}\beta^{\prime \prime }\left(E_{0}\right){\left(E-E_{0}\right)}^{2}+D_{0}\left(\beta_{c}-\beta \right)+\sqrt{2D_{0}}\eta \left(t\right) \end{array}$$

and

(104) $$\begin{array}{c} \frac{dE}{dt}=\frac{1}{2}D_{0}\beta^{\prime \prime }\left(E_{0}\right){\left(E-E_{0}\right)}^{2}+D_{0}\left(\beta_{c}-\beta \right). \end{array}$$

### 7.2. Relaxation Time above the Critical Temperature

We first consider the case $T>T_{c}$ where equilibrium states exist. Close to equilibrium, we can expand the free energy in the Taylor series as $F\left(E\right)=F\left(E_{\mathrm{eq}}\right)+F^{\prime }\left(E_{\mathrm{eq}}\right)\left(E-E_{\mathrm{eq}}\right)+\frac{1}{2}F^{\prime \prime }\left(E_{\mathrm{eq}}\right){\left(E-E_{\mathrm{eq}}\right)}^{2}+\ldots$. Using the fact that $F^{\prime }\left(E_{\mathrm{eq}}\right)=0$ and introducing the squared (effective) pulsation

(105) $$\begin{array}{c} \omega^{2}=F^{\prime \prime }\left(E_{\mathrm{eq}}\right), \end{array}$$

we obtain $F\left(E\right)=F\left(E_{\mathrm{eq}}\right)+\frac{1}{2}\omega^{2}{\left(E-E_{\mathrm{eq}}\right)}^{2}+\ldots$. Therefore, the deterministic Equation (101) becomes

(106) $$\begin{array}{c} \frac{dE}{dt}=-D_{0}\beta \omega^{2}\left(E-E_{\mathrm{eq}}\right). \end{array}$$

Its solution is

(107) $$\begin{array}{c} E\left(t\right)=E_{\mathrm{eq}}+\left[E\left(t=0\right)-E_{\mathrm{eq}}\right]e^{-D_{0}\beta \omega^{2}t}. \end{array}$$

The equilibrium state is stable when $\omega^{2}>0$ and unstable when $\omega^{2}<0$. The relaxation time towards a stable state is $t_{\mathrm{relax}}=1/\left(D_{0}\beta \omega^{2}\right)$.^19^ Close to the critical temperature, using the results from Section 6.1, we find that

(108) $$\begin{array}{c} \omega^{2}=\pm \frac{2}{\beta_{c}}\sqrt{\frac{-\beta^{\prime \prime }\left(E_{0}\right)}{2}}{\left(\beta_{c}-\beta \right)}^{1/2}. \end{array}$$

Therefore, the relaxation time towards the stable state is

(109) $$\begin{array}{c} t_{\mathrm{relax}}=\frac{1}{2D_{0}}\sqrt{\frac{2}{-\beta^{\prime \prime }\left(E_{0}\right)}}{\left(\beta_{c}-\beta \right)}^{-1/2}. \end{array}$$

Introducing the dimensionless variables of Appendix C.1, we obtain

(110) $$\begin{array}{c} t_{\mathrm{relax}}=\frac{1}{6}\sqrt{\frac{2}{-\eta^{\prime \prime }\left(\Lambda_{0}\right)}}{\left(\eta_{c}-\eta \right)}^{-1/2}\frac{t_{K}}{N}\frac{\eta_{c}}{D(\Lambda_{0})}. \end{array}$$

The relaxation time diverges at the critical point as ${\left(\eta_{c}-\eta \right)}^{-1/2}$. The expression of the relaxation time is similar to the prefactor in front of the exponential term in the metastable state lifetime [see Equation (91)]. They differ by a factor π.

### 7.3. Collapse Time below the Critical Temperature

For $T<T_{c}$, there is no equilibrium state, and the system collapses. It ultimately converges towards the condensed state if a small-scale regularization has been accounted for; otherwise, the energy goes to −∞. To study the collapse close to the critical temperature $T_{c}$, we use the normal form of the potential given by Equation (77). The solution of Equation (104) when $T\leq T_{c}$ starting from $E_{0}$ is

(111) $$\begin{array}{c} E\left(t\right)=E_{0}-\sqrt{\frac{2(\beta -\beta_{c})}{-\beta^{\prime \prime }\left(E_{0}\right)}}\tan\left(D_{0}\sqrt{-\frac{1}{2}\beta^{\prime \prime }\left(E_{0}\right)\left(\beta -\beta_{c}\right)}\;t\right). \end{array}$$

The collapse time, corresponding to E→−∞, is given by

(112) $$\begin{array}{c} t_{\mathrm{coll}}=\frac{\pi }{2D_{0}}\sqrt{\frac{2}{-\beta^{\prime \prime }\left(E_{0}\right)}}{\left(\beta -\beta_{c}\right)}^{-1/2}. \end{array}$$

The collapse time diverges when $\beta \to \beta_{c}^{+}$ because the free energy F(E) presents an inflexion point at $E=E_{0}$ when $\beta =\beta_{c}$. Introducing the dimensionless variables of Appendix C.1, we obtain

(113) $$\begin{array}{c} t_{\mathrm{coll}}=\frac{\pi }{6}\sqrt{\frac{2}{-\eta^{\prime \prime }\left(\Lambda_{0}\right)}}{\left(\eta -\eta_{c}\right)}^{-1/2}\frac{t_{K}}{N}\frac{\eta_{c}}{D(\Lambda_{0})}. \end{array}$$

When $\eta >\eta_{c}$, the collapse time diverges at the critical point as $t_{\mathrm{coll}}∼{\left(\eta -\eta_{c}\right)}^{-1/2}$. Interestingly, this is the same scaling as for the divergence of the relaxation (damping) time $t_{\mathrm{relax}}∼{\left(\eta_{c}-\eta \right)}^{-1/2}$ close to the critical point for $\eta <\eta_{c}$ [see Equation (110)]. The prefactors differ by a factor π. As a result, the expression of the collapse time coincides with the prefactor in front of the exponential term in the metastable state lifetime [see Equation (91)].

### 7.4. Finite Size Scaling

In the previous section, we studied the collapse time of a self-gravitating Brownian gas in the weak friction limit when $\eta >\eta_{c}$. For $\eta \to \eta_{c}^{+}$, it is given by Equation (113). This study neglects the fluctuations due to finite *N* effects. As a result, it predicts that the collapse time is infinite when $\eta =\eta_{c}$. Actually, because of fluctuations (finite *N* effects), the collapse time should be large but finite at $\eta =\eta_{c}$. Below, we develop an argument, similar to the one developed in Section VII of [81], to estimate the finite size scaling of the collapse time close to the minimum temperature and its finite value at $\eta =\eta_{c}$.

For $\eta <\eta_{c}$, we have found that the lifetime of a metastable state is given by Equation (91). Finite size effects enter the expression of the metastable state lifetime in the combination $N{\left(\eta_{c}-\eta \right)}^{3/2}$. If we assume that a similar combination enters in the expression of the collapse time for $\eta >\eta_{c}$ we expect a scaling of the form

(114) $$t_{\mathrm{coll}}\propto {\left(\eta -\eta_{c}\right)}^{-1/2}F\left[N{\left(\eta -\eta_{c}\right)}^{3/2}\right]\frac{t_{K}}{N}$$

with F(x)→1 for x→+∞ in order to recover Equation (113) when N→+∞. At $\eta =\eta_{c}$, the singular factor ${\left(\eta -\eta_{c}\right)}^{-1/2}$ must cancel out, implying that $F\left(x\right)∼x^{1/3}$ for x→0. Therefore, at $\eta =\eta_{c}$, the collapse time taking into account fluctuations scales as

(115) $$t_{\mathrm{coll}}\propto N^{1/3}\;\frac{t_{K}}{N},\;\left(\eta =\eta_{c}\right).$$

## 8. Statistical Mechanics of a Binary Star in the Microcanonical Ensemble

To illustrate the nature of phase transitions in self-gravitating systems in a very simple manner, we consider here the statistical mechanics of a binary star [10,15]. It consists of N=2 stars of mass *m* in gravitational interaction. The Hamiltonian is

(116) $$\begin{array}{c} H\left(R,V,r,v\right)=\frac{1}{2}MV^{2}+\frac{1}{2}\mu v^{2}-\frac{Gm^{2}}{r}, \end{array}$$

where (R,V) are the coordinates and momenta of the center of mass and (r,v) are the relative coordinates and momenta of the particles. On the other hand, M=2m is the total mass and μ=m/2 is the reduced mass. We assume that the stars are hard spheres of radius a/2 and that they are confined within a spherical box of radius *R*. Because of the collisions of the particles between themselves and against the boundaries of the box (due to the finite values of *R* and *a*), their phase trajectory is extremely irregular, and this justifies a statistical analysis.^20^ For this two-body system, it is possible to compute the density of states (4) and the partition function (20) exactly analytically [10,15].

In this section, we consider the microcanonical ensemble. To compute the density of states, we introduce the function

(117) $$\begin{array}{c} I\left(E\right)=\int_{H\leq E}\;dRdVdrdv, \end{array}$$

which gives the area of phase space where the energy *H* is less than *E* (action). Integrating over R, we obtain

(118) $$\begin{array}{c} I\left(E\right)=\frac{4}{3}\pi R^{3}\int_{H\leq E}\;dVdrdv. \end{array}$$

The condition H≤E is equivalent to $\left(1/2\right)MV^{2}+\left(1/2\right)\mu v^{2}\leq E+Gm^{2}/r$. If we define $x_{\alpha }={\left(M/2\right)}^{1/2}V_{\alpha }$ for α=1,2,3 and $x_{\alpha }={\left(\mu /2\right)}^{1/2}v_{\alpha }$ for α=4,5,6, the condition H≤E can be rewritten as $\sum_{\alpha =1}^{6}x_{\alpha }^{2}\leq E+Gm^{2}/r$. It characterizes a six-dimensional sphere of “radius” $\sqrt{E+Gm^{2}/r}$. Therefore, we can rewrite Equation (118) as

(119) $$\begin{array}{c} I\left(E\right)=\frac{4}{3}\pi R^{3}{\left(\frac{4}{M\mu }\right)}^{3/2}\int_{\left|x\right|\leq \sqrt{E+Gm^{2}/r}}\;dxdr. \end{array}$$

The integral over x is just the volume of a hypersphere of “radius” $\sqrt{E+Gm^{2}/r}$ in a six-dimensional space. Recalling that $V_{6}=\pi^{3}/6$, we obtain

(120) $$\begin{array}{c} I\left(E\right)=\frac{4}{3}\pi R^{3}{\left(\frac{4}{M\mu }\right)}^{3/2}\frac{\pi^{3}}{6}\int {\left(E+\frac{Gm^{2}}{r}\right)}^{3}\;dr. \end{array}$$

Taking care of the range of integration, we can rewrite the foregoing equation as

(121) $$\begin{array}{c} I\left(E\right)=\frac{64}{9}\pi^{5}\frac{R^{3}}{m^{3}}\int_{a}^{r_{\max}}{\left(E+\frac{Gm^{2}}{r}\right)}^{3}r^{2}\;dr. \end{array}$$

The maximum radius $r_{\max}$ is determined as follows. We must respect the condition $E+Gm^{2}/r\geq 0$. If E≥0, this is always the case, so $r_{\max}=R$. If E<0, we need $r\leq -Gm^{2}/E$. We must therefore compare $-Gm^{2}/E$ and *R*. If $-Gm^{2}/E<R$, i.e., $E<-Gm^{2}/R$, we must take $r_{\max}=-Gm^{2}/E$. If $-Gm^{2}/E>R$, i.e., $-Gm^{2}/R<E<0$, we must take $r_{\max}=R$. We also recall that the minimum energy is $E_{\min}=-Gm^{2}/a$. In conclusion:

(i) If $-Gm^{2}/a<E<-Gm^{2}/R$, we have $r_{\max}=-Gm^{2}/E$.
(ii) If $E>-Gm^{2}/R$, we have $r_{\max}=R$.

The density of states is given by

(122) $$\begin{array}{c} g(E)=\int \delta (H-E)\;dRdVdrdv. \end{array}$$

It satisfies the relation

(123) $$\begin{array}{c} g\left(E\right)=\frac{dI}{dE}. \end{array}$$

From Equation (121), we obtain

(124) $$\begin{array}{c} g\left(E\right)=\frac{64}{3}\pi^{5}\frac{R^{3}}{m^{3}}\int_{a}^{r_{\max}}{\left(E+\frac{Gm^{2}}{r}\right)}^{2}r^{2}\;dr \end{array}$$

or, equivalently,

(125) $$\begin{array}{c} g\left(E\right)=\frac{64}{3}\pi^{5}G^{2}mR^{3}\int_{a}^{r_{\max}}{\left(1+\frac{Er}{Gm^{2}}\right)}^{2}\;dr. \end{array}$$

The integral can be performed analytically, leading to

(126) $$\begin{array}{c} g\left(E\right)=\frac{64}{9}\frac{\pi^{5}G^{3}m^{3}R^{3}}{E}\left[{\left(1+\frac{Er_{\max}}{Gm^{2}}\right)}^{3}-{\left(1+\frac{Ea}{Gm^{2}}\right)}^{3}\right]. \end{array}$$

As discussed previously, we must consider two cases:

(i) If $-Gm^{2}/a<E<-Gm^{2}/R$, we have
  (127) $$\begin{array}{c} g\left(E\right)=\frac{64}{9}\frac{\pi^{5}G^{3}m^{3}R^{3}}{-E}{\left(1+\frac{Ea}{Gm^{2}}\right)}^{3}. \end{array}$$
(ii) If $E>-Gm^{2}/R$, we have
  (128) $$\begin{array}{c} g\left(E\right)=\frac{64}{9}\frac{\pi^{5}G^{3}m^{3}R^{3}}{-E}\left[{\left(1+\frac{Ea}{Gm^{2}}\right)}^{3}-{\left(1+\frac{ER}{Gm^{2}}\right)}^{3}\right]. \end{array}$$

**Remark** **9.** *The quantity I(E) can also be calculated analytically. It can be written as*

(129) $$\begin{array}{c} I\left(E\right)=\frac{64}{9}\pi^{5}G^{3}m^{3}R^{3}\mathrm{sgn}\left(E\right)\int_{\frac{Ea}{Gm^{2}}}^{\frac{Er_{\max}}{Gm^{2}}}{\left(1+x\right)}^{3}\frac{dx}{x} \end{array}$$

*with*

(130) $$\begin{array}{c} \int {\left(1+x\right)}^{3}\frac{dx}{x}=3x+\frac{3}{2}x^{2}+\frac{1}{3}x^{3}+\ln x. \end{array}$$

*We note that I(E) diverges when a=0 while g(E) remains finite. When a→0, we have*

(131) $$\begin{array}{c} I\left(E\right)∼\frac{64}{9}\pi^{5}G^{3}m^{3}R^{3}\ln\mu \end{array}$$

*with μ=R/a. This expression is valid in the dominant approximation μ≫1.*

### 8.1. First Set of Dimensionless Variables

In this subsection, we rewrite the general equations of the binary star problem in the microcanonical ensemble by using the first set of dimensionless variables introduced in Appendix D.1. In terms of these variables, the inverse temperature is given by

(132) $$\begin{array}{c} \frac{1}{t}=\frac{ds}{d\epsilon }\;\mathrm{with}\;s=\ln g\left(\epsilon \right). \end{array}$$

We also introduce the constant $A=\frac{64}{9}\pi^{5}G^{2}mR^{3}a$. We then obtain the following results:

(i) If −1≤ϵ≤−1/μ, we find

(133) $$\begin{array}{c} I\left(\epsilon \right)=A\frac{Gm^{2}}{a}{\left[3x+\frac{3}{2}x^{2}+\frac{1}{3}x^{3}+\ln x\right]}_{-1}^{\epsilon }, \end{array}$$

(134) $$\begin{array}{c} g\left(\epsilon \right)=\frac{A}{-\epsilon }{\left(1+\epsilon \right)}^{3}, \end{array}$$

(135) $$\begin{array}{c} s(\epsilon )=\ln A+3\ln(1+\epsilon )-\ln(-\epsilon ), \end{array}$$

(136) $$\begin{array}{c} \frac{1}{t}=\frac{3}{1+\epsilon }-\frac{1}{\epsilon }. \end{array}$$

We note that these results are independent of μ. The minimum energy, corresponding to the ground state t=0, is $\epsilon_{\min}=-1$. Close to the ground state, we have ϵ≃3t−1 so that C=dϵ/dt=3. On the other hand, the temperature has a local maximum (1/t has a local minimum) at $\epsilon_{*}=-\left(\sqrt{3}-1\right)/2=-0.366025\ldots$ and its value is $t_{*}=\left(2-\sqrt{3}\right)/2=0.133975\ldots$. At that point, the specific heat diverges like $C∼-\frac{\sqrt{3}}{2}{\left(\epsilon -\epsilon_{*}\right)}^{-1}$.

(ii) If ϵ≥−1/μ, we find

(137) $$\begin{array}{c} I\left(\epsilon \right)=A\frac{Gm^{2}}{a}\mathrm{sgn}\left(\epsilon \right){\left[3x+\frac{3}{2}x^{2}+\frac{1}{3}x^{3}+\ln x\right]}_{\epsilon }^{\mu \epsilon }, \end{array}$$

(138) $$\begin{array}{c} g\left(\epsilon \right)=\frac{A}{-\epsilon }\left[{\left(1+\epsilon \right)}^{3}-{\left(1+\mu \epsilon \right)}^{3}\right], \end{array}$$

(139) $$\begin{array}{c} s\left(\epsilon \right)=\ln A+\ln\left[{\left(1+\epsilon \right)}^{3}-{\left(1+\mu \epsilon \right)}^{3}\right]-\ln\left(-\epsilon \right), \end{array}$$

(140) $$\begin{array}{c} \frac{1}{t}=\frac{3\left[{\left(1+\epsilon \right)}^{2}-\mu {\left(1+\mu \epsilon \right)}^{2}\right]}{{\left(1+\epsilon \right)}^{3}-{\left(1+\mu \epsilon \right)}^{3}}-\frac{1}{\epsilon }. \end{array}$$

At high energies, we have the relation $\epsilon =2t-\frac{3}{2}\frac{\mu^{2}-1}{\mu^{3}-1}+\ldots$ so that C=dϵ/dt=2. The temperature has a local minimum (1/t has a local maximum) at

(141) $$\begin{array}{c} \epsilon_{0}=-\frac{3+\sqrt{3}+\left(3-\sqrt{3}\right)\mu }{2(\mu^{2}+\mu +1)},\;t_{c}=\frac{\sqrt{3}\left(\mu -1\right)}{2(\mu^{2}+\mu +1)}. \end{array}$$

For μ→+∞, these quantities behave as $\epsilon_{0}∼-\left(3-\sqrt{3}\right)/\left(2\mu \right)$ and $t_{c}∼\sqrt{3}/\left(2\mu \right)$. When $\epsilon \to \epsilon_{0}$, the specific heat diverges like

(142) $$\begin{array}{c} C∼\frac{\sqrt{3}\left(\mu -1\right)}{2\left(\mu^{2}+\mu +1\right)\left(\epsilon -\epsilon_{0}\right)}. \end{array}$$

The two branches of solution (i) and (ii) connect each other at $\epsilon_{e}=-1/\mu$ and $t_{e}=\left(\mu -1\right)/\left[\left(2+\mu \right)\mu \right]$. At that point, the specific heat is continuous:

(143) $$\begin{array}{c} C_{e}=-\frac{{\left(\mu +2\right)}^{2}}{\mu^{2}-2\mu -2}, \end{array}$$

but its derivative is discontinuous:

(144) $$\begin{array}{c} \frac{dC}{d\epsilon }=\frac{6\mu^{3}\left(\mu +2\right)}{{\left(\mu^{2}-2\mu -2\right)}^{2}}\;\left(\epsilon =\epsilon_{e}^{-}\right), \end{array}$$

(145) $$\begin{array}{c} \frac{dC}{d\epsilon }=\frac{-6\mu^{3}\left(\mu^{3}+3\mu^{2}+3\mu +2\right)}{\left(\mu -1\right){\left(\mu^{2}-2\mu -2\right)}^{2}}\;\left(\epsilon =\epsilon_{e}^{+}\right). \end{array}$$

### 8.2. Second Set of Dimensionless Variables

In this subsection, we rewrite the general equations of the binary star problem in the microcanonical ensemble by using the second set of dimensionless variables introduced in Appendix D.2. In terms of these variables, the inverse temperature is given by

(146) $$\begin{array}{c} \eta =-\frac{dS}{d\Lambda }\;\mathrm{with}\;S=\frac{1}{2}\ln g\left(\Lambda \right). \end{array}$$

We also introduce the constant $K=\mu A=\frac{64}{9}\pi^{5}G^{2}mR^{4}$. This normalization does not depend on *a*, contrary to the previous normalization. We then obtain the following results:

(i) If $1/4\leq \Lambda \leq \Lambda_{\max}=\mu /4$, we find

(147) $$\begin{array}{c} I\left(\Lambda \right)=K\frac{Gm^{2}}{R}{\left[3x+\frac{3}{2}x^{2}+\frac{1}{3}x^{3}+\ln x\right]}_{-1}^{-4\Lambda /\mu }, \end{array}$$

(148) $$\begin{array}{c} g\left(\Lambda \right)=\frac{K}{4\Lambda }{\left(1-\frac{4\Lambda }{\mu }\right)}^{3}, \end{array}$$

(149) $$\begin{array}{c} S\left(\Lambda \right)=\frac{1}{2}\ln K+\frac{3}{2}\ln\left(1-\frac{4\Lambda }{\mu }\right)-\frac{1}{2}\ln\left(4\Lambda \right), \end{array}$$

(150) $$\begin{array}{c} \eta =\frac{6}{\mu -4\Lambda }+\frac{1}{2\Lambda }. \end{array}$$

The minimum energy, corresponding to the ground state η→+∞, is $\Lambda_{\max}=\mu /4$. Close to the ground state, we have Λ≃−3/(2η)+μ/4 so that $C=\eta^{2}d\Lambda /d\eta =3/2$. On the other hand, the temperature presents a local maximum (η presents a local minimum) at $\Lambda_{*}=\frac{\mu }{8}\left(\sqrt{3}-1\right)$, and its value is $\eta_{*}=4/\left[\mu \left(2-\sqrt{3}\right)\right]$. At that point, the specific heat diverges like $C∼\left(\mu \sqrt{3}/16\right){\left(\Lambda -\Lambda_{*}\right)}^{-1}$.

(ii) If Λ≤1/4, we find

(151) $$\begin{array}{c} I\left(\Lambda \right)=K\frac{Gm^{2}}{R}\mathrm{sgn}\left(-\Lambda \right){\left[3x+\frac{3}{2}x^{2}+\frac{1}{3}x^{3}+\ln x\right]}_{-4\Lambda /\mu }^{-4\Lambda }, \end{array}$$

(152) $$\begin{array}{c} g\left(\Lambda \right)=\frac{K}{4\Lambda }\left[{\left(1-\frac{4\Lambda }{\mu }\right)}^{3}-{\left(1-4\Lambda \right)}^{3}\right], \end{array}$$

(153) $$\begin{array}{c} S\left(\Lambda \right)=\frac{1}{2}\ln K+\frac{1}{2}\ln\left[{\left(1-\frac{4\Lambda }{\mu }\right)}^{3}-{\left(1-4\Lambda \right)}^{3}\right]-\frac{1}{2}\ln\left(4\Lambda \right), \end{array}$$

(154) $$\begin{array}{c} \eta =\frac{6}{\mu }\frac{{\left(1-\frac{4\Lambda }{\mu }\right)}^{2}-\mu {\left(1-4\Lambda \right)}^{2}}{{\left(1-\frac{4\Lambda }{\mu }\right)}^{3}-{\left(1-4\Lambda \right)}^{3}}+\frac{1}{2\Lambda }. \end{array}$$

At high energies we have the relation $\Lambda =-1/\eta +\frac{3}{8}\frac{(\mu^{2}-1)\mu }{\mu^{3}-1}+\ldots$ so that $C=\eta^{2}d\Lambda /d\eta =1$. The temperature presents a local minimum (η presents a local maximum) at

(155) $$\begin{array}{c} \Lambda_{0}=\frac{\mu [3+\sqrt{3}+\left(3-\sqrt{3}\right)\mu ]}{8(\mu^{2}+\mu +1)},\;\eta_{c}=\frac{4(\mu^{2}+\mu +1)}{\sqrt{3}\mu \left(\mu -1\right)}. \end{array}$$

For μ→+∞, these quantities tend to $\Lambda_{0}=\left(3-\sqrt{3}\right)/8=0.158494\ldots$ and $\eta_{c}=4/\sqrt{3}=2.3094\ldots$. When $\Lambda \to \Lambda_{0}$, the specific heat diverges like

(156) $$\begin{array}{c} C∼-\frac{\sqrt{3}\left(\mu -1\right)\mu }{16\left(\mu^{2}+\mu +1\right)\left(\Lambda -\Lambda_{0}\right)}. \end{array}$$

The two branches of solution (i) and (ii) connect each other at $\Lambda_{e}=1/4$ and $\eta_{e}=2\left(2+\mu \right)/\left(\mu -1\right)$. At that point, the specific heat is continuous:

(157) $$\begin{array}{c} C_{e}=-\frac{{\left(\mu +2\right)}^{2}}{2(\mu^{2}-2\mu -2)}, \end{array}$$

but its derivative is discontinuous:

(158) $$\begin{array}{c} \frac{dC}{d\Lambda }=-\frac{12\mu^{2}\left(\mu +2\right)}{{\left(\mu^{2}-2\mu -2\right)}^{2}}\;\left(\Lambda =\Lambda_{e}^{+}\right), \end{array}$$

(159) $$\begin{array}{c} \frac{dC}{d\Lambda }=\frac{12\mu^{2}\left(\mu^{3}+3\mu^{2}+3\mu +2\right)}{\left(\mu -1\right){\left(\mu^{2}-2\mu -2\right)}^{2}}\;\left(\Lambda =\Lambda_{e}^{-}\right). \end{array}$$

## 9. Statistical Mechanics of a Binary Star in the Canonical Ensemble

We now consider the statistical mechanics of a binary star in the canonical ensemble. The partition function is given by

(160) $$\begin{array}{c} Z\left(\beta \right)=\int e^{-\beta H}\;dRdVdrdv. \end{array}$$

Integrating over R, V, and v, we obtain

(161) $$\begin{array}{c} Z\left(\beta \right)=\frac{128}{3}\pi^{5}\frac{R^{3}}{{\left(M\mu \right)}^{3/2}\beta^{3}}\int_{a}^{R}e^{\beta Gm^{2}/r}r^{2}\;dr. \end{array}$$

### 9.1. First Set of Dimensionless Variables

Using the first set of dimensionless variables from Appendix D.1, we can rewrite the partition function as

(162) $$\begin{array}{c} Z\left(t\right)=Bt^{3}\int_{1}^{\mu }e^{1/(tx)}x^{2}\;dx \end{array}$$

with $B=\frac{128}{3}\pi^{5}R^{3}m^{3}G^{3}$. The free energy is given by

(163) $$\begin{array}{c} f(t)=-t\ln Z \end{array}$$

and the average energy reads

(164) $$\begin{array}{c} \langle \epsilon \rangle =t^{2}\frac{d\ln Z}{dt}. \end{array}$$

It can be written as

(165) $$\begin{array}{c} \langle \epsilon \rangle =-t^{2}\frac{d}{dt}\left(\frac{f}{t}\right)=-t\frac{df}{dt}+f. \end{array}$$

The integral defining the partition function (162) can be evaluated approximately at high and low temperatures [10]. We can then obtain the free energy and the average energy as a function of the temperature, using Equations (163)–(165).

(i) In the regime of high temperatures (t→+∞), we can make the approximation (see Appendix B)

(166) $$\begin{array}{c} Z\left(t\right)=\frac{1}{3}Bt^{3}\mu^{3}\left(1+\frac{3}{2t\mu }+\ldots \right). \end{array}$$

We then have

(167) $$\begin{array}{c} f\left(t\right)=-3t\ln t-\frac{3}{2\mu }-\left(\ln B+3\ln\mu -\ln3\right)t, \end{array}$$

(168) $$\begin{array}{c} \langle \epsilon \rangle =3t-\frac{3}{2\mu },\;C=3. \end{array}$$

(ii) In the regime of low temperatures (t→0), we can make the approximation (see Appendix B)

(169) $$\begin{array}{c} Z\left(t\right)∼Bt^{4}e^{1/t}\frac{1}{1-2t}. \end{array}$$

We then have

(170) $$\begin{array}{c} f\left(t\right)=-4t\ln t-1-2t^{2}-t\ln B, \end{array}$$

(171) $$\begin{array}{c} \langle \epsilon \rangle =4t-1,\;C=4. \end{array}$$

These two regimes are connected to each other by a plateau. The transition between the two regimes occurs when the free energy has the same value, giving

(172) $$\begin{array}{c} t_{t}≃\frac{1}{3\ln\mu }. \end{array}$$

The variation of energy (latent heat) at the transition is

(173) $$\begin{array}{c} \Delta \langle \epsilon \rangle ≃1-t_{t}. \end{array}$$

**Remark** **10.** *Returning to the original variables, we find that 〈E〉∼3T for T→+∞ and $\langle E\rangle -E_{\min}∼4T$ for T→0. These asymptotic behaviors are similar to those obtained in the microcanonical ensemble where E∼2T for T→+∞ and $E-E_{\min}∼3T$ for T→0. The quantitative difference with the microcanonical description is due to the weak number of particles (N=2). These asymptotic results would coincide in the limit N→+∞.*

### 9.2. Second Set of Dimensionless Variables

Using the second set of dimensionless variables from Appendix D.2, the relation from Equation (25) can be written as

(174) $$\begin{array}{c} \langle \Lambda \rangle =-\frac{d}{d\eta }\left(\eta F\right)=-\eta \frac{dF}{d\eta }-F. \end{array}$$

(i) In the regime of high temperatures (η→0), we have

(175) $$\begin{array}{c} F\left(\eta \right)=-\frac{3}{2\eta }\ln\left(\frac{2}{\mu \eta }\right)-\frac{3}{8}-\frac{1}{2\eta }\left(\ln B+3\ln\mu -\ln3\right), \end{array}$$

(176) $$\begin{array}{c} \langle \Lambda \rangle =-\frac{3}{2\eta }+\frac{3}{8}. \end{array}$$

(ii) In the regime of low temperatures (η→+∞), we have

(177) $$\begin{array}{c} F\left(\eta \right)=-\frac{2}{\eta }\ln\left(\frac{2}{\mu \eta }\right)-\frac{\mu }{4}-\frac{2}{\mu \eta^{2}}-\frac{1}{2\eta }\ln B, \end{array}$$

(178) $$\begin{array}{c} \langle \Lambda \rangle =-\frac{2}{\eta }+\frac{\mu }{4}. \end{array}$$

## 10. Fluctuations of the Energy of a Binary Star in the Canonical Ensemble

The distribution of energies of a binary star at temperature *T* in the canonical ensemble is given by Equation (35) where the free energy F(E) viewed as a thermodynamical potential is defined by Equation (36).

Adopting the first set of dimensionless variables from Appendix D.1, we obtain

(179) $$\begin{array}{c} P\left(\epsilon \right)=\frac{1}{Z(t)}e^{-f(\epsilon )/t} \end{array}$$

with

(180) $$\begin{array}{c} f(\epsilon )=\epsilon -ts(\epsilon ). \end{array}$$

Using the results of Section 8.1, we find that

(181) $$\begin{array}{c} f\left(\epsilon \right)=\epsilon -t\left[\ln A+3\ln(1+\epsilon )-\ln(-\epsilon )\right]\;\left(-1\leq \epsilon \leq -1/\mu \right), \end{array}$$

(182) $$\begin{array}{c} f\left(\epsilon \right)=\epsilon -t\left\{\ln A+\ln\left[{\left(1+\epsilon \right)}^{3}-{\left(1+\mu \epsilon \right)}^{3}\right]-\ln\left(-\epsilon \right)\right\}\;\left(\epsilon \geq -1/\mu \right). \end{array}$$

Adopting the second set of dimensionless variables from Appendix D.2, we obtain

(183) $$\begin{array}{c} P\left(\Lambda \right)=\frac{1}{Z(\eta )}e^{-2\eta F(\Lambda )} \end{array}$$

with

(184) $$\begin{array}{c} F\left(\Lambda \right)=-\Lambda -\frac{1}{\eta }S\left(\Lambda \right). \end{array}$$

Using the results of Section 8.2, we find that

(185) $$\begin{array}{c} F\left(\Lambda \right)=-\Lambda -\frac{1}{2\eta }\left[\ln K+3\ln\left(1-\frac{4\Lambda }{\mu }\right)-\ln\left(4\Lambda \right)\right]\;\left(1/4\leq \Lambda \leq \Lambda_{\max}=\mu /4\right), \end{array}$$

(186) $$\begin{array}{c} F\left(\Lambda \right)=-\Lambda -\frac{1}{2\eta }\left\{\ln K+\ln\left[{\left(1-\frac{4\Lambda }{\mu }\right)}^{3}-{\left(1-4\Lambda \right)}^{3}\right]-\ln\left(4\Lambda \right)\right\}\;\left(\Lambda \leq 1/4\right). \end{array}$$

## 11. Random Transitions of a Binary Star in the Canonical Ensemble

**Figure 3:**
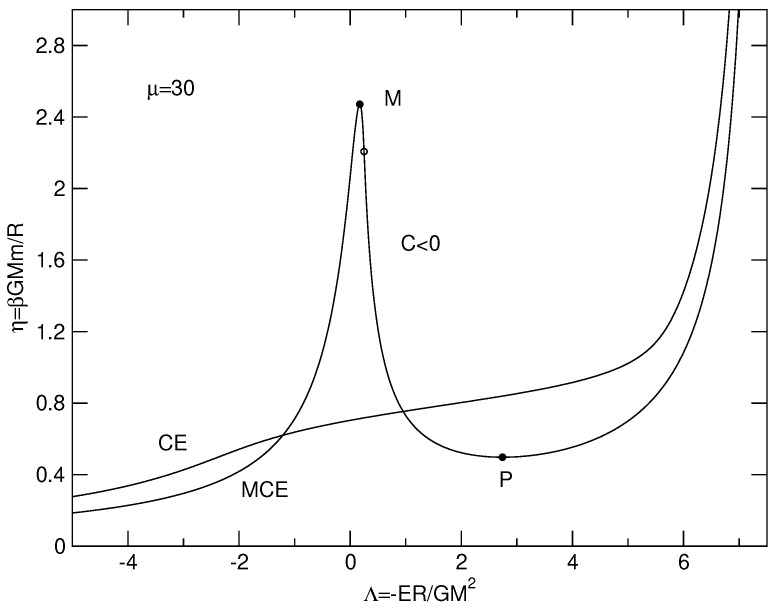
Caloric curves of the two-body system (binary star) in the microcanonical and canonical ensembles. The region of negative specific heat present in the microcanonical ensemble (MCE) is replaced by a first-order phase transition in the canonical ensemble (CE).

The caloric curves of a binary star in the microcanonical and canonical ensembles are represented in Figure 3. We have used the normalization of Appendix D.2. We have adopted a cut-off parameter μ=30 for illustration.

**Figure 4:**
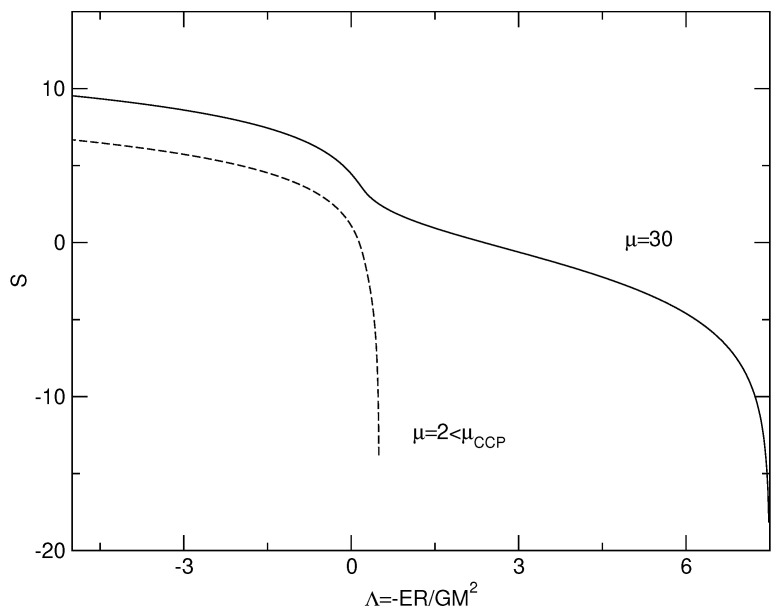
Entropy versus energy plot for μ=30 (microcanonical ensemble). For $\mu >\mu_{\mathrm{CCP}}=1+\sqrt{3}$, the curve presents a convex intruder in the region of negative specific heats. For $\mu \leq \mu_{\mathrm{CCP}}$, the region of negative specific heat disappears and the entropy is concave everywhere.

**Figure 5:**
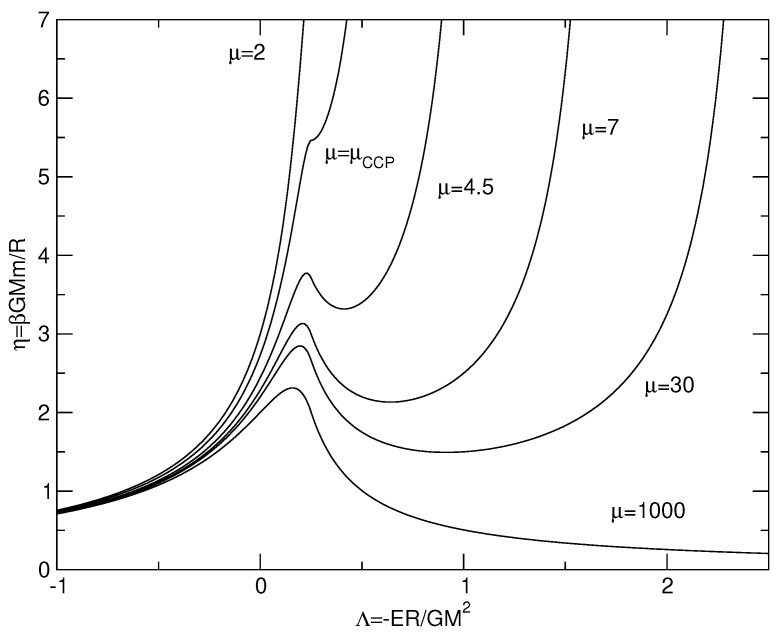
Caloric curves of the two-body system (binary star) in the microcanonical ensemble for various cut-off parameters. The region of negative specific heats and the canonical phase transition are suppressed for $\mu \leq \mu_{\mathrm{CCP}}=1+\sqrt{3}$. Therefore, the binary star model displays a canonical critical point at $\mu =1+\sqrt{3}=2.73205\ldots$, Λ=1/4=0.25 and $\eta =4/[\left(1+\sqrt{3}\right)\left(2-\sqrt{3}\right)]=5.4641\ldots$.

In the microcanonical ensemble, where the control parameter is the energy *E*, the caloric curve is T(E). The minimum energy $E_{\min}=-Gm^{2}/a$ (ground state) is obtained when the two particles are in contact with vanishing velocity. In that case, T=0. At low energies, the particles remain close together on average; this corresponds to the *condensed phase*. When $E\to E_{\min}$, the temperature behaves as $T∼\frac{1}{3}\left(E-E_{\min}\right)$ and the specific heat C=3. At high energies, the typical interparticle distance is of the order of the box radius *R*; this corresponds to the *gaseous phase*. When E→+∞, the temperature behaves as T∼E/2 and the specific heat C=2. At low and high energies, the specific heat is positive because the system feels the influence of the boundaries (the size *a* of the particles or the size *R* of the box). At intermediate energies, between points *M* and *P*, the caloric curve in the microcanonical ensemble displays a region of negative specific heat C<0. The temperature typically behaves as T≃−E which corresponds to a specific heat C=−1. In this range of energies, the system is self-bound and the typical interparticle distance *r* satisfies a≪r≪R. The entropy S(E) is plotted in Figure 4; it presents a convex intruder in the region of negative specific heats (solid line). As we shall see, the region of negative specific heats and the convex intruder in the microcanonical ensemble are replaced by a first-order phase transition in the canonical ensemble. The dependence of the microcanonical caloric curve on the cut-off parameter μ is depicted in Figure 5. For $\mu_{\min}=2\leq \mu \leq \mu_{\mathrm{CCP}}=1+\sqrt{3}$, the region of negative specific heats and the canonical phase transition disappear. This corresponds to a canonical critical point [15].^21^ Below this critical point, the caloric curve is monotonic and the entropy S(E) is concave everywhere (see Figure 4, dashed line). Conversely, for $\mu \geq \mu_{\mathrm{CCP}}=1+\sqrt{3}$, the region of negative specific heat becomes more and more pronounced and extended as μ increases.

**Figure 6:**
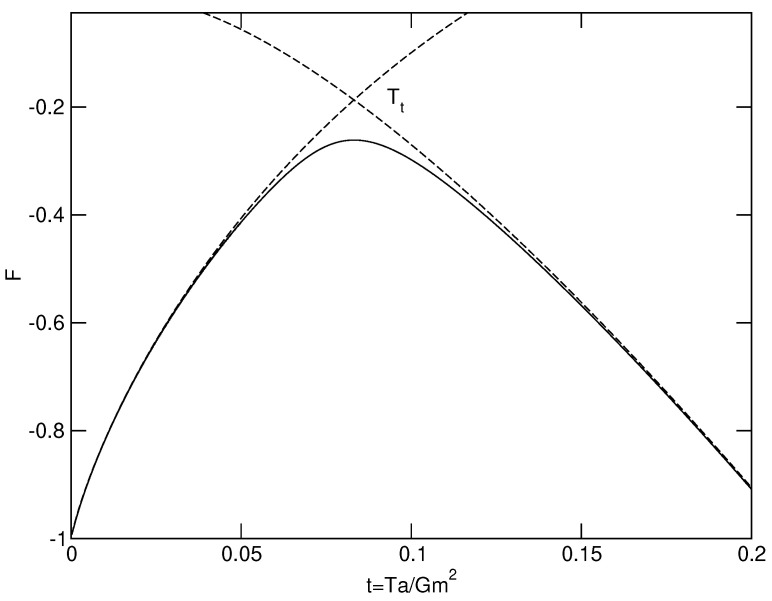
Free energy versus temperature plot for μ=30 (canonical ensemble). We have also indicated the asymptotic limits of the free energy [see Equations (167) and (170)]. This curve displays a first-order phase transition at a typical temperature $t_{t}≃0.0832$ [see Equation (172)].

In the canonical ensemble, where the control parameter is the temperature *T*, the caloric curve is 〈E〉(T). The statistical ensembles are clearly inequivalent. We see in Figure 3 that the caloric curve in the canonical ensemble differs from the caloric curve in the microcanonical ensemble. In particular, the region of negative specific heats allowed in the microcanonical ensemble is replaced by a first-order phase transition in the canonical ensemble corresponding to the isothermal collapse. This phase transition connects the gaseous phase to the condensed phase at a transition temperature $T_{t}$ (see Figure 6). The transition temperature behaves with the cut-off parameter as $T_{t}≃Gm^{2}/\left(3a\ln\left(R/a\right)\right)$ for a≪R. The latent heat released at the transition is $\Delta \langle E\rangle ≃Gm^{2}/a-T_{t}$. More information can be gained by considering metastable equilibrium states as described below.

**Figure 7:**
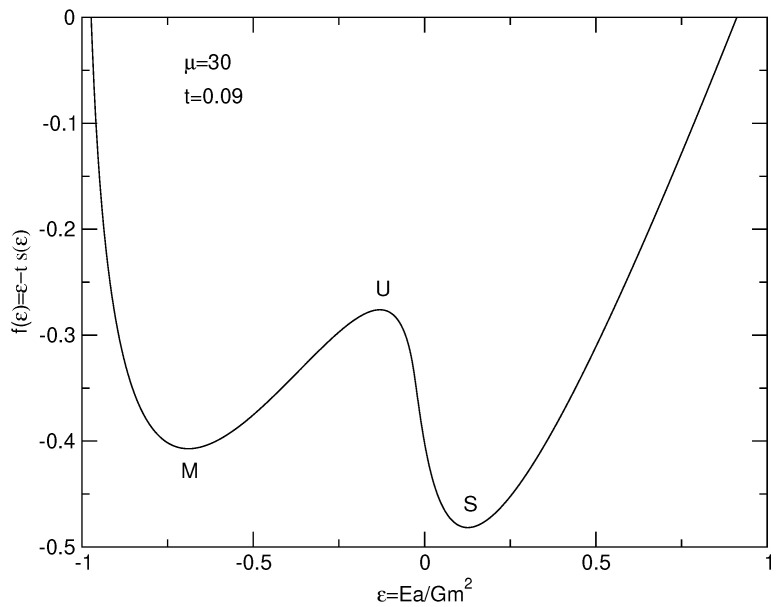
Free energy F(E) of the binary star at fixed temperature *T* interpreted as a thermodynamical potential. It displays two minima (stable and metastable states) separated by a maximum (unstable state).

**Figure 8:**
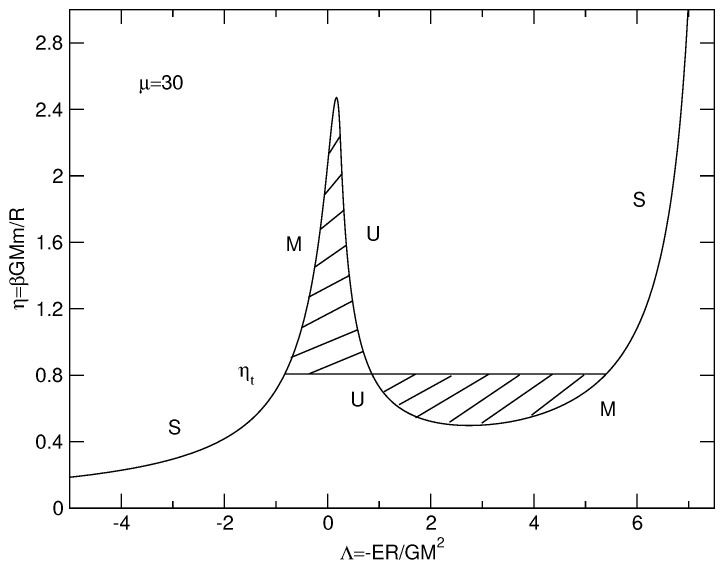
Series of equilibria displaying stable, metastable, and unstable equilibrium states in the canonical ensemble. The transition temperature $T_{t}$ is determined by the Maxwell construction (see [15] for details) or by the equality of the free energy of the gaseous and condensed phases (see Figure 11 below). The physical caloric curve in the canonical ensemble is obtained by keeping stable and metastable states (and discarding unstable states), and the strict caloric curve is obtained by keeping only stable states (see Section 4.3). The strict caloric curve differs from the exact canonical caloric curve of Figure 3 because the number of particles N=2 is small.

**Figure 9:**
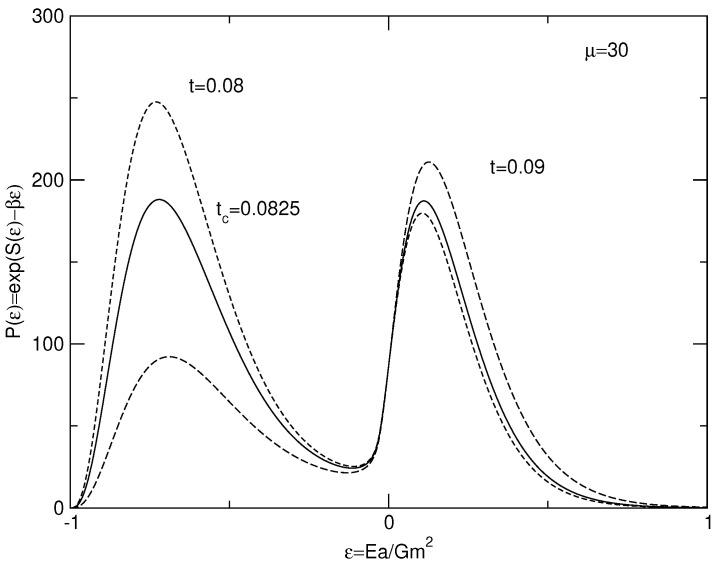
Distribution of energies of the binary star in the canonical ensemble at temperature *T*. In the region of phase transition, the function P(E) has a characteristic bimodal structure. This is characteristic of a first-order phase transition. The most probable energies $E_{\mathrm{cond}}$ and $E_{\mathrm{gas}}>E_{\mathrm{cond}}$ satisfy $F^{\prime }\left(E_{\mathrm{eq}}\right)=0$, i.e., $S^{\prime }\left(E_{\mathrm{eq}}\right)=\beta$. According to Equation (6), they correspond, respectively, to the condensed states and to the gaseous states on the microcanonical curve of Figure 3 (the local minimum of the distribution P(E) corresponds to the equilibrium state with a negative specific heat, which is unstable in the canonical ensemble). At the transition temperature $T_{t}$ the two phases have the same probability (this is only approximate because the bumps are dissymmetrical). Below or above the transition temperature, one of the two phases emerges as the most probable one.

**Figure 10:**
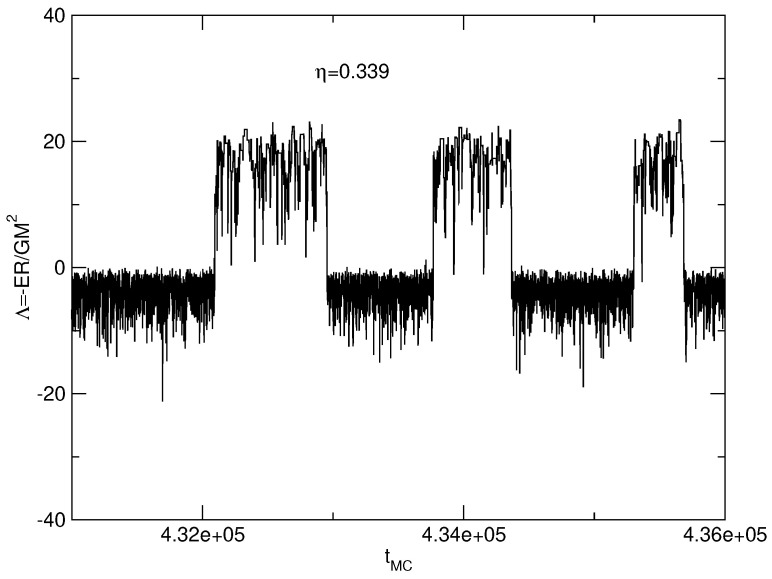
Fluctuations of energy as a function of the Monte Carlo time at the temperature of transition $\eta_{t}=0.339$ for μ=100. The system successively jumps from one phase to the other [15]. This corresponds to a succession of collapses (from the gaseous phase to the condensed phase) and explosions (from the condensed phase to the gaseous phase).

**Figure 11:**
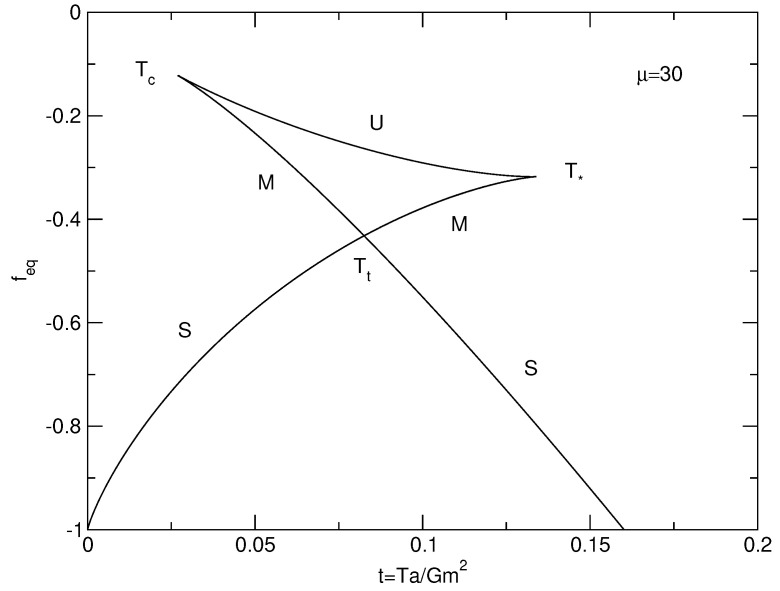
Free energy $F_{\mathrm{eq}}\left(T\right)$ displaying stable, metastable, and unstable equilibrium states. The transition temperature $t_{t}=0.0825$ obtained by this method is close to the exact transition temperature $t_{t}≃0.0832$ from Figure 6 (they would coincide for N→+∞). This figure also shows the barrier of free energy between the metastable state and the unstable state as a function of the temperature.

The fluctuations in the canonical ensemble (fixed temperature *T*) can be studied by considering the free energy F(E) interpreted as a thermodynamical potential. The free energy of the binary star is plotted in Figure 7 for $\mu >\mu_{\mathrm{CCP}}$. Close to the transition temperature $T_{t}$, it displays two minima (stable and metastable states) separated by a maximum (unstable state). Therefore, a free energy barrier separates the two minima of free energy. This is characteristic of a first-order phase transition. These equilibrium states $E_{\mathrm{eq}}\left(T\right)$ can be obtained by rotating the microcanonical caloric curve T(E) from Figure 3 by $90^{o}$. The curve $E_{\mathrm{eq}}\left(T\right)$ defines the series of equilibria in the canonical ensemble displaying stable, metastable, and unstable states (see Figure 8 and Section 4.3).^22^ The distribution of energies P(E) in the canonical ensemble at fixed temperature *T* [see Equation (35)] is represented in Figure 9. It presents two peaks at $E_{\mathrm{cond}}$ and $E_{\mathrm{gas}}>E_{\mathrm{cond}}$ corresponding to the condensed phase and the gaseous phase, respectively. Below or above $T_{t}$, the peaks are dissymmetric and one of the two phases appears to be more probable than the other (the gaseous phase when $T>T_{t}$ and the condensed phase when $T<T_{t}$). However, in practice, the system explores the two phases successively as shown by the Monte Carlo simulation [15] reported in Figure 10. In Figure 11, we have plotted the free energy $F_{\mathrm{eq}}\left(T\right)$ defined in Section 4.3 displaying stable, metastable, and unstable states. This function clearly shows the difference in free energy $\Delta F=F\left(E_{U}\right)-F\left(E_{M}\right)$ between the metastable state and the unstable state at a given temperature. This is the barrier of free energy that the system has to cross to pass from the condensed phase (bound binary star) to the gaseous phase (dissociated binary star) or the converse. At low and high temperatures, there are no metastable states and no random transitions anymore. For $T<T_{c}$, the metastable gaseous phase disappears and the system undergoes a “gravitational collapse” that takes it to the condensed phase. For $T>T_{*}$, the metastable condensed phase disappears and the system undergoes an “explosion”, reverse to the collapse, that takes it to the gaseous phase. In this manner, we can follow a hysteresis cycle in the canonical ensemble by changing the temperature. Similar results have been obtained for a gas of self-gravitating fermions [15] (see Figure 4 in [30]).

It is interesting to specifically consider the case where a=0 (i.e., μ→+∞). We first note that the microcanonical caloric curve tends to a limit when a→0, in which the minimum energy is rejected to infinity ($E_{\min}\to -\infty$) and $T_{*}\to 0$ (see Figure 5). By contrast, the partition function diverges when a→0 so that there is no canonical caloric curve in a strict sense. This is because the partition function is dominated, at any temperature, by the singular condensed state in which the two particles coalesce (r=0). However, gaseous metastable and unstable states exist for $T>T_{c}$. This is revealed by the microcanonical caloric curve rotated by $90^{o}$, which can be interpreted as a series of equilibria in the canonical ensemble (see Section 4.3). This curve displays metastable gaseous states and unstable states. Therefore, in the canonical ensemble, the system may be “blocked” in a gaseous metastable state for some time (see Figure 2). The lifetime of this metastable state with a=0 is determined in Section 12. When the system escapes from this metastable state and forms a singular binary star with r=0, it cannot return to the gaseous state because the free energy of the singular binary star tends to −∞, implying an infinitely large barrier of potential.

**Remark** **11.** In this section, we have considered the case of N=2 self-gravitating Brownian particles. The system displays random transitions associated with a first-order canonical phase transition. For larger values of N, the results of this section remain qualitatively valid, but the lifetime of the metastable states becomes longer and longer, so the random transitions are more and more rare. When N→+∞, the lifetime of the metastable states is infinite, so the first-order phase transition does not take place in practice [15]. There is no random transition. The system remains in the metastable phase until the spinodal points $T_{c}$ and $T_{*}$ at which it experiences a zeroth-order phase transition (collapse and explosion respectively). Furthermore, when N>2 and a is sufficiently small, we can have phase transitions in the microcanonical ensemble (i.e., for isolated systems) corresponding to the gravothermal catastrophe (note that the gravothermal catastrophe does not occur for N=2 particles). This happens when μ=R/a is above the microcanonical critical point $\mu_{\mathrm{MCP}}$ [15]. The previous results remain valid with the roles of energy and the temperature interchanged. For small values of N, the system displays random transitions associated with a first-order microcanonical phase transition. When N is large, the first-order phase transition does not take place in practice because the lifetime of the metastable state tends to infinity [15]. The system remains in the metastable phase until the spinodal points $E_{c}$ and $E_{*}$ at which it experiences a zeroth-order phase transition (collapse and explosion, respectively).

## 12. Lifetime of a Metastable Binary Star with a=0

In this section, we obtain a simple analytical expression for the lifetime of a metastable binary star with a=0 in the canonical ensemble. We first show how the equations of Section 8 and Section 9 simplify when a=0. To that purpose, we use the second normalization^23^ of Appendix D.

### 12.1. Microcanonical Ensemble

In the microcanonical ensemble, the density of states remains finite when a=0. Therefore, there exists a strict equilibrium state for all energies (there is no gravothermal catastrophe when N=2). If we take a=0 in the equations of Section 8, we obtain the following results:

(i) For 1/4≤Λ<+∞, we find

(187) $$\begin{array}{c} g\left(\Lambda \right)=\frac{K}{4\Lambda }, \end{array}$$

(188) $$\begin{array}{c} S\left(\Lambda \right)=\frac{1}{2}\ln K-\frac{1}{2}\ln\left(4\Lambda \right), \end{array}$$

(189) $$\begin{array}{c} \eta =\frac{1}{2\Lambda }, \end{array}$$

(190) $$\begin{array}{c} C=-\frac{1}{2}. \end{array}$$

(ii) For Λ≤1/4, we find

(191) $$\begin{array}{c} g\left(\Lambda \right)=\frac{K}{4\Lambda }\left[1-{\left(1-4\Lambda \right)}^{3}\right], \end{array}$$

(192) $$\begin{array}{c} S\left(\Lambda \right)=\frac{1}{2}\ln K+\frac{1}{2}\ln\left[1-{\left(1-4\Lambda \right)}^{3}\right]-\frac{1}{2}\ln\left(4\Lambda \right), \end{array}$$

(193) $$\begin{array}{c} \eta =-\frac{6{\left(1-4\Lambda \right)}^{2}}{1-{\left(1-4\Lambda \right)}^{3}}+\frac{1}{2\Lambda }, \end{array}$$

(194) $$\begin{array}{c} C=\frac{{\left(3-8\Lambda \right)}^{2}}{6-48\Lambda +64\Lambda^{2}}, \end{array}$$

(195) $$\begin{array}{c} C∼\frac{\sqrt{3}}{16}{\left(\Lambda_{0}-\Lambda \right)}^{-1}\;\left(\Lambda \to \Lambda_{0}\right). \end{array}$$

The caloric curve corresponding to μ→+∞ is represented in Figure 5.

**Remark** **12.** *In terms of the original variables, we have, for $-\infty <E<-Gm^{2}/R$, the particularly simple expressions*

(196) $$\begin{array}{c} g\left(E\right)=\frac{64}{9}\frac{\pi^{5}G^{3}m^{3}R^{3}}{-E}, \end{array}$$

(197) $$\begin{array}{c} S\left(E\right)=\ln\left(\frac{64}{9}\frac{\pi^{5}G^{3}m^{3}R^{3}}{-E}\right), \end{array}$$

(198) $$\begin{array}{c} T(E)=-E, \end{array}$$

(199) $$\begin{array}{c} C=-1. \end{array}$$

*For $E>-Gm^{2}/R$, we have*

(200) $$\begin{array}{c} g\left(E\right)=\frac{64}{9}\frac{\pi^{5}G^{3}m^{3}R^{3}}{-E}\left[1-{\left(1+\frac{ER}{Gm^{2}}\right)}^{3}\right], \end{array}$$

(201) $$\begin{array}{c} S\left(E\right)=\ln\left(\frac{64}{9}\frac{\pi^{5}G^{3}m^{3}R^{3}}{-E}\right)+\ln\left[1-{\left(1+\frac{ER}{Gm^{2}}\right)}^{3}\right], \end{array}$$

(202) $$\begin{array}{c} \frac{1}{T(E)}=-\frac{3R}{Gm^{2}}\frac{{\left(1+\frac{ER}{Gm^{2}}\right)}^{2}}{1-{\left(1+\frac{ER}{Gm^{2}}\right)}^{3}}-\frac{1}{E}, \end{array}$$

(203) $$\begin{array}{c} C=\frac{{\left(3+\frac{2ER}{Gm^{2}}\right)}^{2}}{3+6\frac{ER}{Gm^{2}}+2{\left(\frac{ER}{Gm^{2}}\right)}^{2}}, \end{array}$$

(204) $$\begin{array}{c} C∼\frac{\sqrt{3}}{16}N\frac{GM^{2}}{R}{\left(E-E_{0}\right)}^{-1}\;\left(E\to E_{0}\right). \end{array}$$

### 12.2. Canonical Ensemble

In the canonical ensemble, the partition function diverges when a=0. Therefore, there is no strict equilibrium state in the canonical ensemble. However, there exist metastable gaseous states, as discussed in Section 10 and Section 11. If we take a=0 in the equations of Section 10 we find that the free energy (thermodynamic potential at fixed temperature) is given by

(205) $$\begin{array}{c} F\left(\Lambda \right)=-\Lambda -\frac{1}{2\eta }\left[\ln K-\ln(4\Lambda )\right]\;\left(1/4\leq \Lambda <+\infty \right), \end{array}$$

(206) $$\begin{array}{c} F\left(\Lambda \right)=-\Lambda -\frac{1}{2\eta }\left\{\ln K+\ln\left[1-{\left(1-4\Lambda \right)}^{3}\right]-\ln\left(4\Lambda \right)\right\}\;\left(\Lambda \leq 1/4\right). \end{array}$$

The free energy F(Λ) corresponding to μ→+∞ is represented in Figure 2. It displays a minimum (metastable state) and a maximum (unstable state) forming a barrier of potential. The temporal evolution of the distribution of energy P(E,t) is governed by the Fokker–Planck equation from Equation (59). The normalized diffusion coefficient is given by Equation (A22). We recall that

(207) $$\begin{array}{c} I\left(\Lambda \right)∼\frac{Gm^{2}}{R}K\ln\mu \end{array}$$

in the dominant approximation [see Equation (131)]. Therefore, using Equations (187), (191) and (207), we obtain

(208) $$\begin{array}{c} D(\Lambda )=\Lambda \ln\mu \;(1/4\leq \Lambda <+\infty ), \end{array}$$

(209) $$\begin{array}{c} D\left(\Lambda \right)=\frac{\Lambda \ln\mu }{1-{\left(1-4\Lambda \right)}^{3}}\;\left(\Lambda \leq 1/4\right). \end{array}$$

**Remark** **13.** *In terms of the original variables, we have for $-\infty <E<-Gm^{2}/R$ the particularly simple expressions*

(210) $$\begin{array}{c} F\left(E\right)=E-T\ln\left(\frac{64}{9}\frac{\pi^{5}G^{3}m^{3}R^{3}}{-E}\right), \end{array}$$

(211) $$\begin{array}{c} D(E)=-3MDE\ln\mu . \end{array}$$

*For $E>-Gm^{2}/R$, we have*

(212) $$\begin{array}{c} F\left(E\right)=E-T\ln\left(\frac{64}{9}\frac{\pi^{5}G^{3}m^{3}R^{3}}{-E}\right)-T\ln\left[1-{\left(1+\frac{ER}{Gm^{2}}\right)}^{3}\right], \end{array}$$

(213) $$\begin{array}{c} D\left(E\right)=-3MD\frac{E}{1-{\left(1+\frac{ER}{Gm^{2}}\right)}^{3}}\ln\mu . \end{array}$$

### 12.3. Normal Form of the Potential

Close to the critical point, we find that the normal form of the thermodynamic potential F(Λ) is given by Equation (A31) with

(214) $$\begin{array}{c} \Lambda_{0}=\frac{1}{8}\left(3-\sqrt{3}\right)=0.158494\ldots \end{array}$$

(215) $$\begin{array}{c} \eta_{c}=\frac{4}{\sqrt{3}}=2.3094\ldots \end{array}$$

(216) $$\begin{array}{c} -\eta^{\prime \prime }\left(\Lambda_{0}\right)=\frac{256}{3\sqrt{3}}=49.2672\ldots \end{array}$$

These exact values for N=2 can be compared with the values from Equations (93) and (94) obtained in the mean field limit N→+∞. We can see that they are relatively close to each other. This suggests that the mean field approximation is reasonable even for a small number of particles (see Figure 2).

### 12.4. Kramers Formula

Substituting Equations (214)–(216) into Equation (91), we find that the lifetime of a metastable binary star with a=0 close to the critical point is explicitly given by

(217) $$\begin{array}{c} t_{\mathrm{life}}=0.121816\;\frac{1}{D(\Lambda_{0})}{\left(2.3094-\eta \right)}^{-1/2}e^{0.537285{\left(2.3094-\eta \right)}^{3/2}}t_{K}. \end{array}$$

The effective temperature of collapse $\eta_{l}$ is

(218) $$\begin{array}{c} \eta_{l}=0.796315\ldots \end{array}$$

These exact expressions for N=2 can be compared with the expressions from Equations (95) and (96) based on a mean field approximation:

(219) $$\begin{array}{c} t_{\mathrm{life}}=0.16786975\;\frac{1}{D(\Lambda_{0})}{\left(2.517551-\eta \right)}^{-1/2}e^{0.679192{\left(2.517551-\eta \right)}^{3/2}}t_{K}, \end{array}$$

(220) $$\begin{array}{c} \eta_{l}=1.22334\ldots \end{array}$$

The mean field approximation is reasonable even for N=2 particles.

**Remark** **14.** *More generally, we can use the exact results from Section 12.1 and Section 12.2 to evaluate the barrier of free energy ΔF and the metastable state lifetime $t_{\mathrm{life}}$ from the Kramers formula (88) even if we are not close to the critical point. We can also use the results of Section 8, Section 9 and Section 10 to take into account the finite size a of the particles.*

### 12.5. Particles of Different Mass

The preceding results can be easily generalized in the case where the particles have different masses $m_{1}$ and $m_{2}$. In that case, the total mass is $M=m_{1}+m_{2}$ and the reduced mass is $\mu =m_{1}m_{2}/\left(m_{1}+m_{2}\right)$. We can then easily see that the results of Section 8, Section 9, Section 10, Section 11 and Section 12 remain valid provided that *m* is replaced by $\sqrt{m_{1}m_{2}}$. Then, the lifetime of a metastable binary star with masses $m_{1}$ and $m_{2}$ (and a=0) close to the critical point is given by Equation (217) with

(221) $$\begin{array}{c} \eta =\frac{2\beta Gm_{1}m_{2}}{R}. \end{array}$$

This expression completely determines the exponential term in Equation (217). The expressions of D and $t_{K}$ depend on the diffusion and friction parameters of the two particles (e.g., if they are equal or not), but their values only affect the (unimportant) prefactor in Equation (217).

## 13. Conclusions

In this paper, we have reviewed basic results in the statistical mechanics of self-gravitating systems (and other systems with long-range interactions). We have contrasted the microcanonical ensemble description valid for isolated Hamiltonian systems (e.g., stellar systems) evolving at fixed energy and the canonical ensemble description valid for a dissipative gas of Brownian particles in interaction (e.g., self-gravitating Brownian particles) evolving at fixed temperature. We have recalled that negative specific heats are allowed in the microcanonical ensemble for systems with long-range interactions—and they are usual for self-gravitating systems—while they are forbidden in the canonical ensemble. This is a clear manifestation of ensemble inequivalence for systems with long-range interactions whose energy is nonadditive. In particular, for self-gravitating systems, the region of negative specific heat in the microcanonical ensemble is replaced by a first-order phase transition in the canonical ensemble [10,15,18,19].

We have emphasized the importance of metastable states for systems with long-range interactions. Their lifetime scales as $e^{N}$, making them extremely long-lived when *N* is large [29]. When *N* is small, or if we are sufficiently close to the critical point, their lifetime is reduced and the system may experience random transitions from one metastable state to the other (e.g., between the gaseous phase and the condensed phase). We have illustrated these random phase transitions with a toy model consisting of only N=2 particles in gravitational interaction (“binary star”) where analytical results can be obtained. Preliminary results showing these random transitions were given in [15], and they have been expounded in the present paper. They have been analyzed with the theory developed in [29], which is based on a Fokker–Planck equation in energy space [see Equation (59)]. The lifetime of a metastable state can be calculated by using an adaptation of the Kramers formula [29]. This formula can be obtained in different manners (see Appendix E), either by computing the diffusion current of a stationary state, by solving an eigenvalue equation, or by using the theory of instantons. The random transitions reported in the present paper are similar to those found in other systems with long-range interactions [72,82,83,84,85,86,87]. In the case of self-gravitating Brownian particles in the strong friction limit ξ→+∞, they have been studied in terms of the stochastic Smoluchowski equation from which a Kramers formula can also be derived by using the instanton theory [72,76,84,86,88]. Recently, some authors [89] have studied the lifetime of metastable states near a first-order phase transition in the Thirring model by applying the Fokker–Planck theory developed in [29].

When a=0 and N≫1, the series of equilibria of a self-gravitating system has the form of a spiral. There is no statistical equilibrium state in a strict sense, but long-lived metastable states exist above a minimum energy $E_{c}$ in the microcanonical ensemble or above a minimum temperature $T_{c}$ in the canonical ensemble. Below $E_{c}$, the system undergoes a gravothermal catastrophe [17], leading to a binary star surrounded by a hot halo. Below $T_{c}$, the system undergoes an isothermal collapse [40], leading to a Dirac peak containing all the mass.

When a>0 (e.g., hard spheres, fermions, soften potential…) and N≫1, the spiral unwinds and the collapse stops when the small-scale regularization comes into play. The end-product of the collapse is a “condensate” (e.g., a fermion ball in the case of self-gravitating fermions or a “rocky core” in the case of a hard sphere gas) surrounded by a halo. We can thus describe a phase transition from a gaseous phase unaffected by the small-scale cut-off to a condensed phase resulting from gravitational collapse and dominated by the small-scale cut-off. At high energies or high temperatures, the condensate can experience an explosion, reverse to the collapse, and return to the gaseous phase. Due to the existence of long-lived metastable states, the points of collapse and explosion differ. This leads to a notion of hysteresis cycle in microcanonical and canonical ensembles [15,30,37].

In this paper, we have worked in a box in order to have a well-defined statistical equilibrium state at least in the form of a metastable state. However, in reality, self-gravitating systems are not in boxes! In that case, the statistical mechanics of self-gravitating systems is essentially an out-of-equilibrium problem, and we have to use kinetic theory. The kinetic theory of self-gravitating systems is discussed in [1]. Let us consider the case of stellar systems like globular clusters, which contain a moderate number of stars ($N∼10^{6}$) so that two different regimes can be clearly evidenced on a timescale of the order of the Hubble time (see Appendix D of [2] for more details).

In a first regime, the dynamical evolution of the system is described by the Vlasov–Poisson equations. This is the regime appropriate to elliptical galaxies that contain a large number of stars ($N∼10^{12}$). A self-gravitating system initially out of mechanical equilibrium first undergoes a process of violent collisionless relaxation and achieves a virialized state on a few dynamical times $t_{D}$ [90,91,92]. The inner core is flat (ρ≃cst) and almost isothermal, while the velocity distribution in the envelope is radially anisotropic and the density profile decreases as $\rho ∼r^{-4}$ [90,93,94,95], similarly to Hénon’s isochrone profile [96]. One success of Lynden–Bell’s statistical theory of violent relaxation [92] is that it explains the isothermal core of elliptical galaxies without recourse to collisions. By contrast, the structure of the halo cannot be explained by Lynden–Bell’s theory as it results from an incomplete relaxation. Models of incompletely relaxed stellar systems have been elaborated by different authors [97,98,99]. These theoretical models nicely reproduce the results of observations and numerical simulations [100,101]. In these works, the finite extension of the halo is due to incomplete relaxation. The extension of the halo may also be limited by tidal effects. In that case, the system can be described by a “fermionic” King model justified by the theory of violent relaxation [102].

Then, on a longer (secular) timescale scaling as $t_{\mathrm{coll}}∼\left(N/\ln N\right)t_{D}$, the system undergoes a “collisional” evolution due to finite *N* effects. This evolution can be described by the inhomogeneous Lenard–Balescu equation written with angle-action variables [103,104] or, more simply, by the orbit-averaged Fokker–Planck equation [44,105]. Globular clusters evaporate so they do not reach a statistical equilibrium state in a strict sense (the entropy increases continuously). However, the evaporation of stellar systems is a slow process so that, on intermediate timescales, a globular cluster relaxes through gravitational encounters toward a quasiequilibrium state described by the Michie–King [106,107] distribution which is a truncated Boltzmann distribution taking into account the evaporation of high energy stars. The thermodynamics of tidally truncated self-gravitating systems was studied by Katz [108] and Chavanis et al. [2]. The series of equilibria (caloric curve) is similar to the classical spiral of box-confined stellar systems. The Michie–King distributions correspond to the metastable states studied in a box, except that now the box radius is replaced by the tidal radius beyond which the particles are captured by a neighboring object (e.g., a galaxy in the vicinity of a globular cluster). These quasiequilibrium states are metastable, but they are very long-lived because their lifetime scales like $e^{N}$ (with $N∼10^{6}$ in a globular cluster) [29]. Therefore, these gaseous states can play an important role in the dynamics. Indeed, most stellar systems like globular clusters are in the form of metastable gaseous states described by the Michie–King distribution.

Because of evaporation, the globular clusters slowly lose stars, so their central density increases while their core radius decreases as a result of the virial theorem. In this manner, they follow the series of equilibria—called the King sequence—towards higher and higher central densities. In parallel, the slope of the density profile at large distances $\rho ∼r^{-\alpha (t)}$ decreases (see Figure 20 in [2]). Above a critical density (or below a minimum energy), a globular cluster becomes unstable and undergoes core collapse (gravothermal catastrophe). This corresponds to a critical density slope $\alpha_{\mathrm{MCE}}∼3$ [2]. The system takes a “core–halo” structure. The core, which has a negative specific heat, becomes hotter as it loses energy to the profit of the halo. Therefore, it continues to lose energy and evolves away from equilibrium. By this process, the system becomes hotter and hotter and more and more centrally condensed (as a consequence of the virial theorem). From thermodynamical arguments, the end product of the gravothermal catastrophe is expected to be a binary star with a small mass (2m≪M) but a huge binding energy [44]. The potential energy released by the binary star is redistributed in the halo in the form of kinetic energy (the system heats up), leading to an infinite (very large) entropy. Therefore, core collapse leads to the formation of a binary star surrounded by a hot halo. Cohn [62] studied the dynamical evolution of the gravothermal catastrophe in the microcanonical ensemble (fixed energy) by numerically solving the orbit-averaged Fokker–Planck equation [44,105]. He found that the collapse is self-similar and that the density profile develops a finite-time singularity (core collapse). The central density $\rho_{0}∼{\left(t_{\mathrm{coll}}-t\right)}^{-1.17}$ becomes infinite in a finite time $t_{\mathrm{coll}}$ and the core radius $r_{0}∼{\left(t_{\mathrm{coll}}-t\right)}^{0.53}$ tends to zero, leading to a singular density profile $\rho ∼r^{-2.23}$. We note, however, that the core mass $M_{0}\left(t\right)∼{\left(t_{\mathrm{coll}}-t\right)}^{0.42}$ tends to zero at the collapse time.^24^ Therefore, the divergence of the central density is simply due to a few stars approaching each other. In fact, a binary star is formed in the post-collapse regime and releases so much energy that the halo re-expands in a self-similar manner [61].^25^ Finally, a series of gravothermal oscillations follows [109].

Most globular clusters are close to the limit of microcanonical stability. This is physically natural since the central density increases with time until an instability takes place at a critical value. At that value, the King distribution generates a density profile that is close to the modified Hubble profile with a slope α∼3 (see Figure 18 of [2]). This profile, which was introduced empirically before the King model, gives a good fit to many globular clusters [110]. The fact that it corresponds to the marginal King distribution in the microcanonical ensemble as noted in [2] may explain why it is selected by nature. Therefore, because of close encounters, globular clusters pass from an initial state with α∼4 (violent collisionless relaxation) where the system is similar to Hénon’s isochrone profile to a final state with α∼3 (slow collisional relaxation), where the system is similar to the modified Hubble profile.

We can also apply these results of statistical mechanics to fermionic and bosonic dark matter halos (see Appendix I of [3,111,112]). To some extent, dark matter halos can be described by the King model, which may be justified by a process of violent relaxation coupled with tidal effects [102]. Most dark matter halos are expected to be close to the point of marginal stability. The marginal King density profile, which is flat at the center and decreases as $\rho ∼r^{-3}$ at large distances, is similar to the modified Hubble profile, which is itself relatively similar to the Burkert profile [113] that gives a good fit of many dark matter halos (see Figure 18 of [2] and Figure 1 of [112] for a comparison between these different profiles). Therefore, as suggested in [2,3,111,112], the marginal King profile may justify the empirical Burkert profile of dark matter halos.^26^ If the dark matter halos have a source of “collisionality” (e.g., gravitational encounters or self-interaction), they may evolve along the King sequence and undergo a gravothermal catastrophe at the critical point. If the dark matter halos are not too massive, the gravothermal catastrophe is stopped by quantum mechanics (Pauli’s exclusion principle for fermions or Heisenberg’s uncertainty principle for bosons). This leads to dark matter halos with a “core–halo” structure made of a fermion ball or a bosonic condensate (soliton) surrounded by an approximately isothermal halo or, more realistically, a King density profile [2,3,111,112]. Depending on the characteristics of the dark matter particle, the condensed object may mimic a supermassive black hole at the center of a galaxy or represent a large dark matter bulge (see [111,112] and references therein). If dark matter halos are sufficiently massive, the gravothermal catastrophe may be followed by an instability of general relativistic origin [5,7,115], leading to the formation of a supermassive black hole instead of a fermion ball or a bosonic condensate (soliton). Note that during the gravothermal catastrophe and the gravitational collapse, the envelope remains unaltered. Therefore, the resulting structure is a marginal King profile (with a flat core and a $\rho ∼r^{-3}$ envelope), similar to the Burkert profile, harbouring either a central condensed object (fermion ball or bosonic soliton) or a supermassive black hole. We refer to [111,112,116] and references therein for more details about this scenario (see also the general review [117] on fermionic dark matter).

Finally, we would like to mention that our results concerning the statistical mechanics of N=2 particles in gravitational interaction may find applications in relation to the evolution of primordial black hole binaries during the Big Bang. At that epoch, the Universe was filled with a hot thermal plasma, and black holes may have appeared in it out of gravitational instabilities, eventually forming binaries.

## Data Availability

Data are contained within the article.

## Notes

1 The literature on the subject is vast. A detailed historical account can be found in the introductions of our papers [1,2,3,4,5,6,7,8], where an exhaustive list of references is given. We also refer to the reviews [9,10,11,12,13,14,15] on the statistical mechanics of self-gravitating systems and to the book [16] on the dynamics and thermodynamics of systems with long-range interactions.

2 For self-gravitating systems and other systems with long-range interactions, negative specific heats are allowed in the microcanonical ensemble, whereas they are forbidden in the canonical ensemble. This implies that the statistical ensembles may be inequivalent in some range of energy and temperature [17,18,19]. Therefore, a clear distinction must be made between microcanonical and canonical ensembles. The region of negative specific heat in the microcanonical ensemble is replaced by an isothermal phase transition in the canonical ensemble

3 Padmanabhan [10] and Campa et al. [16] argue that only the microcanonical ensemble makes sense for systems with long-range interactions. Although we agree on this point for what concerns *isolated* Hamiltonian systems with long-range interactions, we argued in [15,20,21] that the canonical ensemble can describe a *dissipative* gas of Brownian particles with long-range interactions in contact with a thermal bath.

4 The self-gravitating Brownian gas may describe the evolution of planetesimals in the solar nebula in the context of planet formation. In that case, the dust particles experience friction with the gas and a stochastic force due to Brownian motion or turbulence [23]. If the gas of dust particles is sufficiently dense (e.g., at special locations such as large-scale vortices), then self-gravity becomes important, leading to gravitational collapse and planet formation. The canonical (and grand canonical) ensemble may also describe an isothermal self-gravitating gas such as the interstellar medium in equilibrium with a radiation background that imposes the temperature at 3K in regions devoid of heating sources [24].

5 A small-scale regularization may also be introduced by taking quantum effects into account, e.g., by invoking the Pauli exclusion principle for fermions [15,25,26,27,28].

6 The extrema of entropy at fixed mass and energy coincide with the extrema of free energy at fixed mass (first variations of the thermodynamic potential). Therefore, the series of equilibria is the same in the two ensembles. Ensemble inequivalence may occur when we consider the stability of the system (second variations of the thermodynamic potential).

7 When quantum mechanics is taken into account, the collapse stops when the system feels the effect of the Pauli exclusion principle. The resulting equilibrium state is a “fermion ball”, resembling a white dwarf star at zero temperature, surrounded by a halo [15,25,26,27,28]. In the core, further gravitational contraction is prevented by the quantum pressure. We can thus describe a phase transition from a weakly inhomogeneous gaseous phase to a condensed phase with a core–halo structure. At high energies in the microcanonical ensemble or at high temperatures in the canonical ensemble, the condensate experiences an explosion, reverse to the collapse, and returns to the gaseous phase. Due to the existence of long-lived metastable states, the points of collapse and explosion differ. This leads to a notion of a hysteresis cycle [15,30]. Similar results are obtained for a hard sphere gas and for other forms of small-scale regularization [28,31,32,33,34,35,36,37,38,39].

8 Throughout this paper, we take the Boltzmann constant $k_{B}=1$.

9 If the system is confined within a box or surrounded by an external medium, we need to take into account pressure forces in the virial theorem and the specific heat may be positive.

10 A similar mechanism is at work in globular clusters and leads to a gravothermal catastrophe [17].

11 The final equilibrium state of the system is just two stars in Keplerian orbits, all the other stars being dispersed to infinity. In this sense, the statistical mechanics of self-gravitating systems is essentially an out-of-equilibrium problem. However, the evaporation rate is small in general, and the system can be found in a quasiequilibrium state for a relatively long time (see the conclusion).

12 We can also invoke quantum mechanics. For self-gravitating fermions, the small-scale stabilization is due to the Pauli exclusion principle, and for self-gravitating bosons in the form of Bose-Einstein condensates (BECs), it is due to the Heisenberg uncertainty principle (see, e.g., [48]).

13 The entropy is usually written as S=lnW, where *W* is the number of microstates (complexions) associated with a given macrostate. This is the usual manner to compute the entropy. This relation is referred to as the Boltzmann formula, although Boltzmann never wrote this formula explicitly (this relation was formulated by Planck [49]). Conversely, assuming that the entropy is known, Einstein [50] wrote this relation under the form $P\left[f\right]=e^{S[f]}$, where P[f] is the probability density of the macrostate *f* (Einstein interpreted it as the probability of a fluctuation). The density of states is therefore the integral of P[f] over *f*, taking into account the constraints E[f]=E and M[f]=M.

14 A summary of the Poincaré turning point criterion for linear series of equilibria is given in Appendix C of [5].

15 In the present context, the canonical distribution is justified from the *N*-body Fokker–Planck equation, not by considering a subpart of a large isolated system. As we have already indicated, this last procedure is not valid for a Hamiltonian system whose energy is non-additive. By contrast, our justification of the canonical ensemble for a Brownian system whose dynamics is described by a set of Langevin equations is completely rigorous even if the particles have long-range interactions. It is also valid for an arbitrary number of particles, even for only N=2 Brownian particles in interaction.

16 This relation was first established by Gibbs [73] and re-discovered by Einstein [74] (see Pais [75], p. 874).

17 Another possibility is to invoke quantum mechanics and study a gas of self-gravitating Brownian fermions as discussed in [28,30].

18 In principle, we should integrate over the momenta $p_{i}$ rather than over the velocities $v_{i}$ (see Appendix A) but, in the present context, we find it more convenient to work in terms of the velocity.

19 In the unstable case, $\gamma =1/(D_{0}\beta |\omega^{2}|)$ represents the growth rate of the instability.

20 This statistical analysis is rigorously justified for self-gravitating Brownian particles described by the Langevin Equations (16) in the canonical ensemble even for N=2 particles.

21 At the canonical critical point $\mu_{\mathrm{CCP}}=1+\sqrt{3}=2.73205\ldots$, the specific heat diverges at $E_{0}=E_{*}=E_{e}=-\left(\sqrt{3}-1\right)Gm^{2}/\left(2a\right)=-0.366025\ldots Gm^{2}/a$, corresponding to $T_{c}=T_{*}=T_{e}=\left(2-\sqrt{3}\right)Gm^{2}/\left(2a\right)=0.133975\ldots Gm^{2}/a$. It behaves as $C∼\left(\sqrt{3}/2\right)\left(Gm^{2}/a\right){\left(E_{e}-E\right)}^{-1}$ for $E<E_{e}$ and as $C∼\left(\sqrt{3}-1\right)/\left[2\left(\sqrt{3}+1\right)\right]\left(Gm^{2}/a\right){\left(E-E_{e}\right)}^{-1}$ for $E>E_{e}$.

22 The strict caloric curve $E_{\mathrm{strict}}\left(T\right)$ in the canonical ensemble obtained by keeping only the global minima of free energy F(E) differs from the exact caloric curve 〈E〉(T) because of finite *N* effects (here N=2). They coincide when N→+∞.

23 We cannot use the first normalization because the size *a* of the particles explicitly appears in the scales used to normalize the variables.

24 Previously, Larson [58], Hachisu et al. [59] and Lynden–Bell and Eggleton [60] studied the same problem by using fluid equations and obtained similar results.

25 Heggie and Stevenson [63] confirmed these results by constructing self-similar solutions of the orbit-averaged-Fokker–Planck equation in the pre-collapse and post-collapse regimes.

26 The Burkert profile is similar to the Navarro–Frenk–White (NFW) profile [114] at large distances but presents a central core instead of a $r^{-1}$ cusp in agreement with the observations.

## Appendix A. Density of States in d Dimensions

We can easily generalize the results of Section 5.1 in *d* dimensions. The hypervolume of phase space with energy less than *E* is

(A1) $$\begin{array}{c} I\left(E\right)=\int_{H\leq E}\prod_{i=1}^{N}dr_{i}dp_{i} \end{array}$$

and the density of states is

(A2) $$\begin{array}{c} g\left(E\right)=\frac{dI}{dE}=\int \delta \left(E-H\right)\prod_{i=1}^{N}dr_{i}dp_{i}, \end{array}$$

where p=mv is the momentum. Proceeding as in Section 5.1, we obtain

(A3) $$\begin{array}{c} I\left(E\right)={\left(2m\right)}^{dN/2}V_{dN}\int {\left(E-U\right)}^{dN/2}\prod_{i=1}^{N}dr_{i} \end{array}$$

and

(A4) $$\begin{array}{c} g\left(E\right)=\frac{dN}{2}{\left(2m\right)}^{dN/2}V_{dN}\int {\left(E-U\right)}^{dN/2-1}\prod_{i=1}^{N}dr_{i}. \end{array}$$

In d=1 dimension and for N=1 particle in a potential U(x), the foregoing equations reduce to

(A5) $$\begin{array}{c} I\left(E\right)=2\sqrt{2m}\int {\left(E-U\right)}^{1/2}\;dx, \end{array}$$

(A6) $$\begin{array}{c} g\left(E\right)=\sqrt{2m}\int \frac{1}{{\left(E-U\right)}^{1/2}}\;dx. \end{array}$$

Recalling that $E=p^{2}/2m+U$, they can be rewritten as

(A7) $$\begin{array}{c} I(E)=2\int p\;dx=\oint p\;dx, \end{array}$$

(A8) $$\begin{array}{c} g\left(E\right)=\oint \frac{dx}{v}=\frac{1}{\Omega (E)}=\frac{dI}{dE}. \end{array}$$

We see that *I* corresponds to the action of the particle, and g(E) is the inverse of its pulsation Ω(E)=dE/dI.

## Appendix B. Asymptotic Behavior of the Partition Function of the Binary Star

For N=2 particles in gravitational interaction, the partition function is given by [see Equation (162)]

(A9) $$\begin{array}{c} Z\left(t\right)=Bt^{3}\int_{1}^{\mu }e^{1/(tx)}x^{2}\;dx. \end{array}$$

It can be rewritten as

(A10) $$\begin{array}{c} Z\left(t\right)=Bt^{3}\int_{1}^{\mu }e^{\Phi_{t}\left(x\right)}\;dx, \end{array}$$

where we have introduced the function $\Phi_{t}\left(x\right)=2\ln x+\frac{1}{tx}$. This function tends to +∞ for x→0 and x→+∞ and presents a minimum at $x_{m}=1/\left(2t\right)$ whose value is $\Phi_{t}\left(x_{m}\right)=2-2\ln\left(2t\right)$. We also have $\Phi_{t}\left(1\right)=1/t$ and $\Phi_{t}\left(\mu \right)=2\ln\mu +\frac{1}{t\mu }$.

For t→+∞ (high temperatures), we can expand the exponential term in Taylor series, and we obtain

(A11) $$\begin{array}{c} Z\left(t\right)=Bt^{3}\int_{1}^{\mu }\left(1+\frac{1}{tx}+\ldots \right)x^{2}\;dx. \end{array}$$

After integration, we find that

(A12) $$\begin{array}{c} Z\left(t\right)=\frac{1}{3}Bt^{3}\mu^{3}\left(1+\frac{3}{2t\mu }+\ldots \right), \end{array}$$

where we have assumed μ≫1 to simplify some terms.

For t→0 (low temperatures), the integral is dominated by the behavior of $\Phi_{t}\left(x\right)$ close to x=1. Replacing this function by its expansion in Taylor series, we obtain

(A13) $$\begin{array}{c} Z\left(t\right)∼Bt^{3}\int_{1}^{\mu }e^{\frac{1}{t}+\left(2-\frac{1}{t}\right)\left(x-1\right)}\;dx. \end{array}$$

After integration, we find that

(A14) $$\begin{array}{c} Z\left(t\right)∼Bt^{4}e^{1/t}\frac{1}{1-2t}. \end{array}$$

## Appendix C. Reformulation of the Main Results in Terms of Dimensionless Variables

It is useful to introduce dimensionless variables in the equations of Section 6 in order to highlight the scaling of the different quantities with the number of particles *N*.

### Appendix C.1. General Dimensionalization

We introduce the dimensionless variables

(A15) $$\begin{array}{c} \Lambda =-E=-\frac{ER}{GM^{2}},\;\eta =\frac{1}{T}=\frac{\beta GMm}{R}, \end{array}$$

(A16) $$\begin{array}{c} S=\frac{S}{N},\;F=\frac{FR}{GM^{2}}. \end{array}$$

The relation (6) between the inverse temperature and the energy can be written as

(A17) $$\begin{array}{c} \eta =\frac{dS}{dE}. \end{array}$$

The specific heat from Equation (9) can be written as

(A18) $$\begin{array}{c} C=NC\;\mathrm{with}\;C=\frac{dE}{dT}=\eta^{2}\frac{d\Lambda }{d\eta }. \end{array}$$

The free energy from Equation (36) can be written as

(A19) $$\begin{array}{c} \beta F=N\eta F\;\mathrm{with}\;F(E)=E-TS. \end{array}$$

From the original *N*-body Kramers Equation (18), we have the scalings $t∼v^{2}/D∼1/\xi$ with ξ=Dβm (Einstein relation). Therefore, we can define the Kramers relaxation time by

(A20) $$\begin{array}{c} t_{K}=\frac{1}{\xi }=\frac{T}{Dm}. \end{array}$$

This friction or diffusion timescale is independent of *N*. On the other hand, we can write the energy-dependent diffusion coefficient (60) as

(A21) $$\begin{array}{c} D\left(E\right)=3MD\frac{GM^{2}}{R}D\left(E\right) \end{array}$$

with

(A22) $$\begin{array}{c} D\left(E\right)=\frac{\frac{I(E)}{g(E)}}{\frac{GM^{2}}{R}}. \end{array}$$

Combining Equation (A20) with Equation (A21), we obtain

(A23) $$\begin{array}{c} D\left(E\right)=3N\frac{T}{t_{K}}\frac{GM^{2}}{R}D\left(E\right). \end{array}$$

Finally, the lifetime of a metastable state [see Equation (69)] can be written as

(A24) $$\begin{array}{c} t_{\mathrm{life}}=\frac{\pi }{3}\sqrt{C_{M}\left|C_{U}\right|}\frac{t_{K}}{N\eta }\frac{1}{D(E_{U})}e^{N\eta \Delta F}. \end{array}$$

This formula clearly shows that for systems with long-range interactions (e.g., self-gravitating systems), the lifetime of a metastable state scales as $e^{N}$ [29].

### Appendix C.2. Normal Form of the Thermodynamic Potential Close to the Critical Temperature

The Formula (A24) giving the lifetime of a metastable state can be simplified close to the critical point by using the normal (saddle-node) form of the thermodynamic potential. In terms of the dimensionless variables introduced in Appendix C.1, the thermodynamic potential (free energy) is given by

(A25) $$\begin{array}{c} F\left(\Lambda \right)=-\Lambda -\frac{1}{\eta }S\left(\Lambda \right). \end{array}$$

The equilibrium states correspond to $F^{\prime }\left(\Lambda_{\mathrm{eq}}\right)=0$, yielding

(A26) $$\begin{array}{c} \eta =-S^{\prime }\left(\Lambda_{\mathrm{eq}}\right). \end{array}$$

Close to the critical point $\eta_{c}$ (minimum temperature), where $\eta^{\prime }\left(\Lambda_{0}\right)=0$, we can make the expansion (up to second order):

(A27) $$\begin{array}{c} \eta =\eta_{c}+\frac{1}{2}\eta^{\prime \prime }\left(\Lambda_{0}\right){\left(\Lambda_{\mathrm{eq}}-\Lambda_{0}\right)}^{2}+\ldots \end{array}$$

On the other hand, expanding F(Λ) close to $\Lambda_{0}$, we obtain (up to third order):

(A28) $$\begin{array}{c} F\left(\Lambda \right)=-\Lambda_{0}-\frac{1}{\eta }S\left(\Lambda_{0}\right)+\left[-1-\frac{1}{\eta }S^{\prime }\left(\Lambda_{0}\right)\right]\left(\Lambda -\Lambda_{0}\right)-\frac{1}{2\eta }S^{\prime \prime }\left(\Lambda_{0}\right){\left(\Lambda -\Lambda_{0}\right)}^{2}\\ -\frac{1}{6\eta }S^{\prime \prime \prime }\left(\Lambda_{0}\right){\left(\Lambda -\Lambda_{0}\right)}^{3}+\ldots \end{array}$$

Using Equation (A26), we find that

(A29) $$\begin{array}{c} S^{\prime }\left(\Lambda_{0}\right)=-\eta_{c},\;S^{\prime \prime }\left(\Lambda_{0}\right)=-\eta^{\prime }\left(\Lambda_{0}\right)=0,\;S^{\prime \prime \prime }\left(\Lambda_{0}\right)=-\eta^{\prime \prime }\left(\Lambda_{0}\right). \end{array}$$

Therefore, we can rewrite Equation (A28) as

(A30) $$\begin{array}{c} F\left(\Lambda \right)=-\Lambda_{0}-\frac{1}{\eta }S\left(\Lambda_{0}\right)+\left(-1+\frac{\eta_{c}}{\eta }\right)\left(\Lambda -\Lambda_{0}\right)+\frac{1}{6}\frac{\eta^{\prime \prime }\left(\Lambda_{0}\right)}{\eta }{\left(\Lambda -\Lambda_{0}\right)}^{3}. \end{array}$$

Since $\eta ≃\eta_{c}$, we can make the additional approximation

(A31) $$\begin{array}{c} F\left(\Lambda \right)=F_{0}+\frac{\eta_{c}-\eta }{\eta_{c}}\left(\Lambda -\Lambda_{0}\right)+\frac{1}{6}\frac{\eta^{\prime \prime }\left(\Lambda_{0}\right)}{\eta_{c}}{\left(\Lambda -\Lambda_{0}\right)}^{3}, \end{array}$$

where $F_{0}=-\Lambda_{0}-\frac{1}{\eta_{c}}S\left(\Lambda_{0}\right)$. We can check that the condition $F^{\prime }\left(\Lambda_{\mathrm{eq}}\right)=0$ returns Equation (A27). Equation (A27) can be inverted to give

(A32) $$\begin{array}{c} \Lambda_{\mathrm{eq}}=\Lambda_{0}\pm \sqrt{\frac{2}{-\eta^{\prime \prime }\left(\Lambda_{0}\right)}}{\left(\eta_{c}-\eta \right)}^{1/2}. \end{array}$$

Using Equation (A31), we find that the barrier of free energy between the metastable state and the unstable state is given by

(A33) $$\begin{array}{c} \Delta F=\frac{4}{3\eta_{c}}\sqrt{\frac{2}{-\eta^{\prime \prime }\left(\Lambda_{0}\right)}}{\left(\eta_{c}-\eta \right)}^{3/2}. \end{array}$$

The specific heat can be written as

(A34) $$\begin{array}{c} C=\eta^{2}\frac{d\Lambda }{d\eta }. \end{array}$$

Using Equation (A32) we obtain close to the critical point

(A35) $$\begin{array}{c} C=\pm \frac{\eta_{c}^{2}}{2}\sqrt{\frac{2}{-\eta^{\prime \prime }\left(\Lambda_{0}\right)}}{\left(\eta_{c}-\eta \right)}^{-1/2} \end{array}$$

or, equivalently,

(A36) $$\begin{array}{c} C=\frac{\eta_{c}^{2}}{-\eta^{\prime \prime }\left(\Lambda_{0}\right)}{\left(\Lambda_{0}-\Lambda_{\mathrm{eq}}\right)}^{-1}. \end{array}$$

These equations can also be directly derived from the relation [see Equation (41)]

(A37) $$\begin{array}{c} F^{\prime \prime }\left(\Lambda_{\mathrm{eq}}\right)=\frac{\eta }{C} \end{array}$$

by using Equation (A31).

### Appendix C.3. Lifetime of Metastable States

Close to the critical point, Equation (A24) can be rewritten in a sufficient approximation as

(A38) $$\begin{array}{c} t_{\mathrm{life}}=\frac{\pi }{3}\sqrt{C_{M}\left|C_{U}\right|}\frac{t_{K}}{N\eta_{c}}\frac{1}{D(\Lambda_{0})}e^{N\eta_{c}\Delta F}. \end{array}$$

Substituting Equations (A33) and (A35) into Equation (A38), we find that the lifetime of a metastable state close to the critical point is given by

(A39) $$\begin{array}{c} t_{\mathrm{life}}=\frac{\pi }{3}\frac{\eta_{c}}{\sqrt{-2\eta^{\prime \prime }\left(\Lambda_{0}\right)}}\frac{1}{D(\Lambda_{0})}{\left(\eta_{c}-\eta \right)}^{-1/2}e^{\frac{4}{3}N\sqrt{\frac{2}{-\eta^{\prime \prime }\left(\Lambda_{0}\right)}}{\left(\eta_{c}-\eta \right)}^{3/2}}\frac{t_{K}}{N}. \end{array}$$

We can estimate the effective temperature of collapse $\eta_{l}$ due to finite *N* effects by writing

(A40) $$\begin{array}{c} \frac{4}{3}N\sqrt{\frac{2}{-\eta^{\prime \prime }\left(\Lambda_{0}\right)}}{\left(\eta_{c}-\eta_{l}\right)}^{3/2}∼1. \end{array}$$

This gives

(A41) $$\begin{array}{c} \eta_{l}=\eta_{c}\left[1-{\left(\frac{3}{4}\right)}^{2/3}\frac{1}{\eta_{c}}{\left(\frac{-\eta^{\prime \prime }\left(\Lambda_{0}\right)}{2}\right)}^{1/3}N^{-2/3}\right]. \end{array}$$

## Appendix D. Dimensionalization for a Binary Star

In the study of the statistical mechanics of a binary star performed in Section 8, Section 9, Section 10, Section 11 and Section 12 it is convenient to introduce two sets of dimensionless variables.

### Appendix D.1. First Set of Dimensionless Variables

We introduce the dimensionless variables

(A42) $$\begin{array}{c} \epsilon =\frac{Ea}{Gm^{2}},\;t=\frac{Ta}{Gm^{2}},\;\mu =\frac{R}{a}, \end{array}$$

(A43) $$\begin{array}{c} s=S,\;f=\frac{aF}{Gm^{2}},\;C=\frac{d\epsilon }{dt}. \end{array}$$

The normalization scales involve the size *a* of the particles.

### Appendix D.2. Second Set of Dimensionless Variables

For N=2, the general dimensionless variables of Appendix C.1 become

(A44) $$\begin{array}{c} \Lambda =-E=-\frac{ER}{4Gm^{2}},\;\eta =\frac{1}{T}=\frac{2\beta Gm^{2}}{R}, \end{array}$$

(A45) $$\begin{array}{c} S=\frac{S}{2},\;F=\frac{FR}{4Gm^{2}},\;C=\frac{dE}{dT}=\eta^{2}\frac{d\Lambda }{d\eta }. \end{array}$$

The normalization scales do not involve the size *a* of the particles. We have the following relations between the two sets of variables:

(A46) $$\begin{array}{c} \eta =\frac{2}{t\mu },\;\Lambda =-\frac{\epsilon \mu }{4},\;S=\frac{s}{2},\;F=\frac{f\mu }{4},\;C=\frac{C}{2}. \end{array}$$

We also write

(A47) $$\begin{array}{c} A=\frac{K}{\mu }\;\mathrm{with}\;K=\frac{64}{9}\pi^{5}G^{2}mR^{4}. \end{array}$$

In this manner, the constant *K* does not depend on the size *a* of the particles, and we can use these variables even when a=0 (see Section 12).

## Appendix E. Kramers Formula

In this Appendix, we review different methods to derive the Kramers formula, and we discuss their connections.

### Appendix E.1. Stationary Solution of the Smoluchowski Equation

We consider an overdamped Brownian particle (or an ensemble of noninteracting overdamped Brownian particles) in a one-dimensional potential U(x). The evolution of its probability density P(x,t) to be at *x* at time *t* is governed by the Smoluchowski equation

(A48) $$\begin{array}{c} \frac{\partial P}{\partial t}=\frac{\partial }{\partial x}\left[D\left(x\right)\left(\frac{\partial P}{\partial x}+\beta mP\frac{dU}{dx}\right)\right]. \end{array}$$

The term in brackets is minus the diffusion current:

(A49) $$\begin{array}{c} J\left(x,t\right)=-D\left(x\right)\left(\frac{\partial P}{\partial x}+\beta mP\frac{dU}{dx}\right). \end{array}$$

We assume that the potential U(x) has a local minimum at A, a maximum at *B* and a global minimum at *C* (or no global minimum at all). We want to determine the typical time taken by the Brownian particle initially at *A* to cross the barrier of potential. This represents the lifetime of the metastable state. If the height of the potential barrier is large compared to the energy of the thermal motions (mΔU/T≫1), we can assume that the system is approximately in statistical equilibrium but that there is a small current of diffusion J→0, allowing it to cross the barrier of potential. We look for a stationary solution of Equation (A48) corresponding to ∂P/∂t=0 so that *J* is constant. This leads to the differential equation

(A50) $$\begin{array}{c} \frac{dP}{dx}+\beta mP\frac{dU}{dx}=-\frac{J}{D(x)}. \end{array}$$

Its general solution is

(A51) $$P\left(x\right)=P\left(x_{B}\right)e^{-\beta m\left[U\left(x\right)-U\left(x_{B}\right)\right]}+Je^{-\beta mU(x)}\int_{x}^{x_{B}}\frac{e^{\beta mU(y)}}{D(y)}\;dy.$$

Applying this expression at $x=x_{A}$, we obtain

(A52) $$J=\frac{P\left(x_{A}\right)e^{\beta mU(x_{A})}-P\left(x_{B}\right)e^{\beta mU(x_{B})}}{\int_{x_{A}}^{x_{B}}\frac{e^{\beta mU(x)}}{D(x)}\;dx}.$$

Since $P(x_{B})≃0$, we find that

(A53) $$J=\frac{P\left(x_{A}\right)e^{\beta mU(x_{A})}}{\int_{x_{A}}^{x_{B}}\frac{e^{\beta mU(x)}}{D(x)}\;dx}.$$

Close to $x≃x_{A}$, the system is described in good approximation by the Boltzmann distribution

(A54) $$P\left(x\right)≃\frac{e^{-\beta mU(x)}}{\int_{-\infty }^{+\infty }e^{-\beta mU(x)}\;dx}.$$

Applying this formula at $x=x_{A}$ and substituting the resulting expression into Equation (A53), we obtain

(A55) $$J=\frac{1}{\int_{-\infty }^{+\infty }e^{-\beta mU(x)}\;dx\int_{x_{A}}^{x_{B}}\frac{e^{\beta mU(x)}}{D(x)}\;dx}.$$

For $x≃x_{A}$ we can expand the potential up to second order as $U\left(x\right)=U\left(x_{A}\right)+\frac{1}{2}\omega_{A}^{2}{\left(x-x_{A}\right)}^{2}+\ldots$ with $\omega_{A}^{2}=U^{\prime \prime }\left(x_{A}\right)>0$. Therefore, in a good approximation, we have

(A56) $$\int_{-\infty }^{+\infty }e^{-\beta mU(x)}\;dx=e^{-\beta mU(x_{A})}\int_{-\infty }^{+\infty }e^{-\beta m\omega_{A}^{2}x^{2}/2}\;dx=e^{-\beta mU(x_{A})}{\left(\frac{2\pi }{\beta m\omega_{A}^{2}}\right)}^{1/2}.$$

Similarly, for $x≃x_{B}$, we can make the Taylor expansion $U\left(x\right)=U\left(x_{B}\right)+\frac{1}{2}\omega_{B}^{2}{\left(x-x_{B}\right)}^{2}+\ldots$ with $\omega_{B}^{2}=U^{\prime \prime }\left(x_{B}\right)<0$. This gives, in a good approximation,

(A57) $$\begin{array}{c} \int_{x_{A}}^{x_{B}}\frac{e^{\beta mU(x)}}{D(x)}\;dx=\frac{e^{\beta mU(x_{B})}}{D(x_{B})}\int_{-\infty }^{0}e^{\beta m\omega_{B}^{2}x^{2}/2}\;dx=\frac{e^{\beta mU(x_{B})}}{2D(x_{B})}{\left(\frac{-2\pi }{\beta m\omega_{B}^{2}}\right)}^{1/2}. \end{array}$$

Substituting these expressions into Equation (A53), we obtain

(A58) $$J=\frac{\beta m\omega_{A}\left|\omega_{B}\right|}{\pi }D\left(x_{B}\right)e^{-\beta m\Delta U},$$

where $\Delta U=U\left(x_{B}\right)-U\left(x_{A}\right)$ is the barrier of potential, and we recall that

(A59) $$\begin{array}{c} \omega_{A}^{2}=U^{\prime \prime }\left(x_{A}\right)>0,\;\omega_{B}^{2}=U^{\prime \prime }\left(x_{B}\right)<0 \end{array}$$

are the squared pulsations about the metastable and unstable states. When *D* is constant, using the Einstein relation

(A60) $$\begin{array}{c} D=\frac{T}{\xi m}, \end{array}$$

we recover the original Kramers formula

(A61) $$J=\frac{\omega_{A}\left|\omega_{B}\right|}{\pi \xi }e^{-\beta m\Delta U}.$$

On the other hand, since $P(x_{B})≃0$, we obtain from Equation (A51) the distribution

(A62) $$P\left(x\right)=Je^{-\beta mU(x)}\int_{x}^{x_{B}}\frac{e^{\beta mU(y)}}{D(y)}\;dy.$$

This can be seen as a truncated Boltzmann distribution that vanishes when $x=x_{B}$ (we take P(x)=0 for $x\geq x_{B}$). The normalization condition $\int_{-\infty }^{x_{B}}P\left(x\right)\;dx=1$ yields the refined expression of the current

(A63) $$J=\frac{1}{\int_{-\infty }^{x_{B}}dx\;e^{-\beta mU(x)}\int_{x}^{x_{B}}\frac{e^{\beta mU(y)}}{D(y)}\;dy}.$$

If we replace $x_{B}$ by +∞ in the first integral of the denominator and *x* by $x_{A}$ in the second integral of the denominator, we recover Equation (A55).

**Remark** **A1.**
*Some authors, including Kramers [80], assume that the point B where $P(x_{B})=0$ is located substantially after the local maximum of the potential. In that case, we have to integrate from −∞ to +∞ in Equation (A57). Consequently, we have to divide Equation (A61) by 2.*

### Appendix E.2. Eigenvalue Equation

Looking for a solution of the Smoluchowski Equation (A48) of the form

(A64) $$\begin{array}{c} P\left(x,t\right)=Ae^{-\lambda t}g\left(x\right), \end{array}$$

we obtain the eigenvalue equation

(A65) $$\begin{array}{c} \frac{d}{dx}\left[D\left(x\right)\left(\frac{dg}{dx}+\beta mg\frac{dU}{dx}\right)\right]=-\lambda g. \end{array}$$

It can be rewritten as

(A66) $$\begin{array}{c} \frac{d}{dx}\left[D\left(x\right)e^{-\beta mU}\frac{d}{dx}\left(e^{\beta mU}g\right)\right]=-\lambda g. \end{array}$$

Integrating this equation between −∞ and *x*, we obtain

(A67) $$\begin{array}{c} D\left(x\right)e^{-\beta mU}\frac{d}{dx}\left(e^{\beta mU}g\right)=-\lambda \int_{-\infty }^{x}g\left(y\right)\;dy. \end{array}$$

A second integration between $x_{A}$ and *x* gives

(A68) $$g\left(x\right)=e^{-\beta mU(x)}\left\{g\left(x_{A}\right)e^{\beta mU(x_{A})}-\lambda \int_{x_{A}}^{x}dx^{\prime }\;\frac{e^{\beta mU(x^{\prime })}}{D(x^{\prime })}\int_{-\infty }^{x^{\prime }}g\left(y\right)\;dy\right\}.$$

For a large barrier of potential (mΔU/T≫1), we expect that λ→0. At leading order, we have

(A69) $$g_{0}\left(x\right)=g_{0}\left(x_{A}\right)e^{-\beta m(U\left(x\right)-U\left(x_{A}\right))}\;\mathrm{and}\;\lambda_{0}=0.$$

Substituting this leading order term in the right-hand side of Equation (A68), we obtain the expression of g(x) valid up to the first order in λ:

(A70) $$\begin{array}{c} g\left(x\right)=e^{-\beta m(U\left(x\right)-U\left(x_{A}\right))}g_{0}\left(x_{A}\right)\left\{1-\lambda \int_{x_{A}}^{x}dx^{\prime }\;\frac{e^{\beta mU(x^{\prime })}}{D(x^{\prime })}\int_{-\infty }^{x^{\prime }}e^{-\beta mU(y)}\;dy\right\}. \end{array}$$

Writing $g(x_{B})=0$, we obtain the fundamental eigenvalue

(A71) $$\begin{array}{c} \lambda =\frac{1}{\int_{x_{A}}^{x_{B}}dx\;\frac{e^{\beta mU(x)}}{D(x)}\int_{-\infty }^{x}e^{-\beta mU(y)}\;dy}. \end{array}$$

If we extend the second integral in Equation (A71) to +∞, we obtain

(A72) $$\begin{array}{c} \lambda =\frac{1}{\int_{x_{A}}^{x_{B}}dx\;\frac{e^{\beta mU(x)}}{D(x)}\int_{-\infty }^{+\infty }e^{-\beta mU(x)}\;dx}, \end{array}$$

and we recover the Kramers Formula (A55). We also have

(A73) $$\begin{array}{c} g\left(x\right)=e^{-\beta m(U\left(x\right)-U\left(x_{A}\right))}g_{0}\left(x_{A}\right)\left\{1-\lambda \int_{x_{A}}^{x}dy\;\frac{e^{\beta mU(y)}}{D(y)}\int_{-\infty }^{+\infty }e^{-\beta mU(x)}\;dx\right\}, \end{array}$$

which coincides with Equation (A62).

### Appendix E.3. Instanton Theory

The Kramers formula can also be derived from the instanton theory as detailed in [72,86,118].

### Appendix E.4. Relation between J and λ

Let us show the connection between the current *J* and the eigenvalue λ. The mass of the system at time *t* is

(A74) $$\begin{array}{c} M\left(t\right)=M_{0}\int_{-\infty }^{x_{B}}P\left(x,t\right)\;dx, \end{array}$$

where $M_{0}$ is its initial mass (for convenience, we consider here the case of an ensemble of noninteracting Brownian particles instead of just one test particle). Taking the time derivative of Equation (A74) and using the Smoluchowski Equation (A48), we obtain

(A75) $$\begin{array}{c} \frac{dM}{dt}=M_{0}\int_{-\infty }^{x_{B}}\frac{\partial P}{\partial t}\left(x,t\right)\;dx=-M_{0}J\left(x_{B},t\right). \end{array}$$

On the other hand, the mass decreases as

(A76) $$\begin{array}{c} M\left(t\right)=M_{0}e^{-\lambda t}. \end{array}$$

Therefore,

(A77) $$\begin{array}{c} \frac{dM}{dt}=-\lambda M_{0}e^{-\lambda t}. \end{array}$$

Equating Equations (A75) and (A77), we find that

(A78) $$\begin{array}{c} \lambda e^{-\lambda t}=J\left(x_{B},t\right). \end{array}$$

For λ→0, this relation reduces to λ=J. Therefore, the current *J* is equivalent to the eigenvalue λ. They are equal to the inverse of the lifetime of the metastable state:

(A79) $$\begin{array}{c} \lambda =J=\frac{1}{t_{\mathrm{life}}}. \end{array}$$

**Remark** **A2.**
*Using $P(x_{B})=0$ in Equation (A49), we can rewrite Equation (A75) as*

(A80)
$$\begin{array}{c} \frac{dM}{dt}=M_{0}D\left(x_{B}\right)\left(\frac{\partial P}{\partial x}\right)\left(x_{B},t\right). \end{array}$$
